# Immune and endothelial activation markers and risk stratification of childhood pneumonia in Uganda: A secondary analysis of a prospective cohort study

**DOI:** 10.1371/journal.pmed.1004057

**Published:** 2022-07-13

**Authors:** Chloe R. McDonald, Aleksandra Leligdowicz, Andrea L. Conroy, Andrea M. Weckman, Melissa Richard-Greenblatt, Michelle Ngai, Clara Erice, Kathleen Zhong, Sophie Namasopo, Robert O. Opoka, Michael T. Hawkes, Kevin C. Kain

**Affiliations:** 1 SAR Laboratories, Sandra Rotman Centre for Global Health, University Health Network-Toronto General Hospital, Toronto, Canada; 2 Department of Medicine, Division of Critical Care Medicine, Robarts Research Institute, University of Western Ontario, London, Ontario, Canada; 3 Department of Pediatrics, Indiana University, School of Medicine, Indianapolis, Indiana, United States of America; 4 Department of Laboratory Medicine and Pathobiology, University of Toronto, Toronto, Canada; 5 Public Health Ontario Laboratory, Toronto, Canada; 6 Department of Paediatrics, Kabale Regional Referral Hospital, Kabale, Uganda; 7 Department of Paediatrics and Child Health, Mulago Hospital and Makerere University, Kawempe, Kampala, Uganda; 8 Division of Pediatric Infectious Diseases, University of Alberta, Edmonton, Canada; 9 Toronto General Hospital Research Institute, University Health Network, Toronto, Canada; 10 Department of Medicine, University of Toronto, Toronto, Canada; 11 Tropical Disease Unit, Division of Infectious Diseases, Department of Medicine, University of Toronto, Toronto, Canada; Makerere University Medical School, UGANDA

## Abstract

**Background:**

Despite the global burden of pneumonia, reliable triage tools to identify children in low-resource settings at risk of severe and fatal respiratory tract infection are lacking. This study assessed the ability of circulating host markers of immune and endothelial activation quantified at presentation, relative to currently used clinical measures of disease severity, to identify children with pneumonia who are at risk of death.

**Methods and findings:**

We conducted a secondary analysis of a prospective cohort study of children aged 2 to 59 months presenting to the Jinja Regional Hospital in Jinja, Uganda between February 2012 and August 2013, who met the Integrated Management of Childhood Illness (IMCI) diagnostic criteria for pneumonia. Circulating plasma markers of immune (IL-6, IL-8, CXCL-10/IP-10, CHI3L1, sTNFR1, and sTREM-1) and endothelial (sVCAM-1, sICAM-1, Angpt-1, Angpt-2, and sFlt-1) activation measured at hospital presentation were compared to lactate, respiratory rate, oxygen saturation, procalcitonin (PCT), and C-reactive protein (CRP) with a primary outcome of predicting 48-hour mortality. Of 805 children with IMCI pneumonia, 616 had severe pneumonia. Compared to 10 other immune and endothelial activation markers, sTREM-1 levels at presentation had the best predictive accuracy in identifying 48-hour mortality for children with pneumonia (AUROC 0.885, 95% CI 0.841 to 0.928; *p* = 0.03 to *p* < 0.001) and severe pneumonia (AUROC 0.870, 95% CI 0.824 to 0.916; *p* = 0.04 to *p* < 0.001). sTREM-1 was more strongly associated with 48-hour mortality than lactate (AUROC 0.745, 95% CI 0.664 to 0.826; *p* < 0.001), respiratory rate (AUROC 0.615, 95% CI 0.528 to 0.702; *p* < 0.001), oxygen saturation (AUROC 0.685, 95% CI 0.594 to 0.776; *p* = 0.002), PCT (AUROC 0.650, 95% CI 0.566 to 0.734; *p* < 0.001), and CRP (AUROC 0.562, 95% CI 0.472 to 0.653; *p* < 0.001) in cases of pneumonia and severe pneumonia. The main limitation of this study was the unavailability of radiographic imaging.

**Conclusions:**

In this cohort of Ugandan children, sTREM-1 measured at hospital presentation was a significantly better indicator of 48-hour mortality risk than other common approaches to risk stratify children with pneumonia. Measuring sTREM-1 at clinical presentation may improve the early triage, management, and outcome of children with pneumonia at risk of death.

**Trial registration:**

The trial was registered at clinicaltrial.gov (NCT 04726826).

## Abstract 2

Chloe R. McDonald and colleagues assess the ability of immune and endothelial activation markers to identify risk of death from pneumonia among children in Uganda.

## Author summary

**Why was this study done?:**

- Pneumonia remains a leading cause of child mortality, especially in low-resource settings.
- Despite this disease burden, we have few objective triage tools to enable the early recognition and treatment of children with pneumonia at risk of severe and fatal disease.

**What did the researchers do and find?:**

- The study examined whether markers of immune and endothelial activation (e.g., sTREM-1) measured at presentation are better at identifying children with pneumonia who are at risk of death than other current approaches (e.g., respiratory rate, peripheral oxygen saturation, lactate, or acute phase proteins).
- In this study of 805 Ugandan children with pneumonia, sTREM-1 measured at hospital presentation was a significantly better predictor of children at risk of 48-hour and in-hospital death than respiratory rate, peripheral oxygen saturation, lactate, procalcitonin (PCT), or C-reactive protein (CRP).

**What do these findings mean?:**

- Measuring circulating markers with pathobiological links to severe infection (e.g., sTREM-1) at presentation may help to risk stratify children with pneumonia.
- This approach could enhance the early recognition, management, and outcome for high-risk children, while reducing unnecessary hospital admission and over-administration of antibiotics in children with mild and self-limited pneumonia.

## Introduction

Pneumonia is a leading cause of child mortality [1]. In 2016, pneumonia resulted in an estimated 920,000 deaths in children under the age of 5 [2]. Despite significant progress in reducing mortality in children under 5, deaths resulting from pneumonia are not decreasing at the same rate as deaths from other diseases, especially in low-resource settings [3]. Risk factors for pediatric pneumonia are closely tied to socioeconomic status, malnutrition, crowding, lack of immunization, indoor air pollution, low rates of exclusive breastfeeding, and importantly, a lack of access to prompt diagnosis and appropriate treatment [4]. Consequently, the burden of severe and fatal pneumonia is greatest in resource-constrained settings [5,6].

The principal focus of global efforts is to reduce pneumonia deaths in children under 5 [1]. This requires early triage and treatment of children with impending critical illness. However, few prognostic tools are currently available to recognize children at risk of severe and fatal pneumonia [7]. Moreover, the performance of currently utilized triage tools, such as lactate, respiratory rate (RR), and oxygen saturation (SpO_2_), as well as host acute phase markers (e.g., procalcitonin (PCT) and C-reactive protein (CRP)), have not been well studied as predictors of outcome in children with pneumonia in low-resource settings [8].

The diagnosis of childhood pneumonia in low-resource settings is based on criteria set by the World Health Organization (WHO) Integrated Management of Childhood Illness (IMCI) and Integrated Community Case Management [9]. Once pneumonia is diagnosed, the recommended treatment is a 3-day course of oral amoxicillin. However, the IMCI diagnosis of pneumonia relies on the presenting respiratory rate that has multiple limitations [10]. Fast breathing is neither a sensitive nor a specific symptom and its application to the diagnosis of childhood pneumonia results in antibiotic overuse [11]. Access to radiographic imaging, a reference standard in the diagnosis of pneumonia, is lacking in most low-resource settings. This barrier combined with limited access to trained healthcare workers in community settings impedes the rapid identification and triage of children with pneumonia at risk of life-threatening infection.

Better triage tools to enable timely and accurate risk stratification of pediatric pneumonia may decrease mortality rates. Immune and endothelial activation are key contributors to the pathogenesis of severe infection [12–16]. Elevated circulating markers of these pathways are early indicators of severe infection that precede the loss of endothelial integrity that contributes to disease progression, end-organ dysfunction, and death [13,14,17–19]. Mediators involved in immune and endothelial activation are independent and quantitative predictors of disease severity and outcome in many life-threatening infections [13,20,21] and could enable early triage of pediatric pneumonia.

Our objective was to examine the ability of circulating markers of immune and endothelial activation to identify children with IMCI-defined pneumonia who are at risk of death. We hypothesized that biomarkers with pathobiological links to severe infection would be better able to identify children with pneumonia at risk of death relative to currently used clinical features (e.g., respiratory rate, oxygen saturation) and nonspecific reference circulating markers of shock (e.g., lactate) and inflammation (e.g., PCT, CRP).

## Methods

### Patient selection

The study participants included a subset of children who were enrolled in a prospective observational cohort of children aged 2 to 59 months presenting to the Jinja Regional Referral Hospital with a febrile syndrome between February 2012 and August 2013, as described [21–23]. A prespecified analysis of the main prospective observational cohort included comparison of biomarkers in children with pneumonia (S1 Protocol). Inclusion criteria for the main study included: age 2 to 59 months, parental report of fever within the last 48 hours or axillary temperature greater than 37.5°C, hospitalization according to the admitting physician’s judgment [21]. Patients were managed in accordance with national standard of care for the treatment of malaria, pneumonia, sepsis, meningitis, respiratory distress, anemia, and hypoglycemia. Clinical investigations at the time of hospital admission included peripheral oxygen saturation, malaria diagnosis based on blood microscopy and 3-band rapid diagnostic malaria test, glucose, lactate, hemoglobin, and a rapid HIV test [23]. Pneumonia was classified according to the WHO IMCI definition [9] and included: a history of cough or difficulty breathing, and if present, respiratory rate >50 breaths/minute if <12 months or >40 breaths/minute if ≥12 months old. Severe pneumonia was defined as presence of pneumonia and any general danger sign, including nasal flaring, subcostal retractions, convulsions, Blantyre Coma Scale (BCS) <5 or “alert, voice, pain, unresponsive” (AVPU) scale less than “alert,” or the inability to drink or feed. The accompanying caregiver provided informed written consent. Ethical approval was obtained from the Ugandan National Council for Science and Technology, Makerere University Research Ethics Committee in Uganda (Kampala, Uganda, REC Protocol # REF 2011–255) and the University Health Network (Toronto, Canada, REB 12-0039-AE). The trial was registered at clinicaltrial.gov (NCT 04726826).

This study is reported as per the “Reporting recommendations for tumor marker prognostic studies” (REMARK) checklist for observational studies of prognostic markers (S1 Checklist).

### Plasma analyte quantification

Markers of immune and endothelial activation were quantified in EDTA-anticoagulated plasma collected at hospital presentation and stored at −80°C, without a freeze-thaw, until assayed. The Luminex multiplex platform with reagents from R&D Systems [22] was used to quantify 6 markers of immune activation and 5 markers of endothelial activation. Markers of immune activation included: interleukin-6 (IL-6), interleukin-8 (IL-8), interferon-gamma-inducible protein-10/c motif chemokine 10 (IP-10/CXCL-10), chitinase-3-like-1 protein (CHI3L1), soluble tumor necrosis factor receptor-1 (sTNFR-1), soluble triggering receptor expressed on myeloid cells-1 (sTREM-1), PCT, and CRP. Markers of endothelial activation included: soluble vascular cell adhesion molecule (sVCAM-1), soluble intracellular adhesions molecule-1 (sICAM-1), angiopoietin-1 (Angpt-1), angiopoietin-2 (Angpt-2), and soluble fms-like tyrosine kinase-1 (sFlt-1). Protein concentrations below the limit of detection were assigned a value of one third of the lowest point on the standard curve, and assays were performed blinded to the study endpoint.

### Statistical analysis

The study design was outlined as part of secondary analysis planned for the main cohort (S1 Protocol), and statistical tests were selected prior to data analysis. Only children with complete outcome data and an available plasma sample were included in the analysis (S1 Fig). Statistical analysis was performed using STATA v12 (StataCorp, TX) and R v3.2.1 (R Foundation for Statistical Computing) software. Our primary outcome was in-hospital mortality before 48 hours. We also assessed all in-hospital mortality. Descriptive data were summarized using median (interquartile range, IQR) or n (%) and compared using a Wilcoxon rank-sum test or chi-squared test where appropriate. Single and multiple variable logistic regression for the outcomes of 48-hour in-hospital mortality and all in-hospital mortality were performed. Prognostic accuracy for each outcome was assessed by the area under the receiver operating characteristic curve (AUROC) using log-transformed circulating marker concentrations. Univariate and multivariable models that included protein markers and clinical parameters relative to the strongest biomarker model were compared with AUROC model comparison after Bonferroni correction.

## Results

### Study cohort

A total of 2,502 children were enrolled in the parent study [21], 2,084 of whom had a plasma sample available at the time of hospital presentation and complete follow-up to hospital discharge (S1 Fig). Of the children with complete follow-up, 805 (39%) met the IMCI diagnostic criteria for pneumonia, of whom 616 met criteria for severe pneumonia. Demographics and clinical data at the time of hospital presentation are presented in Table 1.

**Table 1:** Patient characteristics.

	n	IMCI pneumonia (*n* = 805)	n	Severe pneumonia (*n* = 616)
**Demographics** ^1^				
Age (months)	804	16 [9, 24]	615	15 [8, 24]
Age <12 months	804	312 (38.8)	615	257 (41.7)
Sex (female)	798	360 (44.7)	610	287 (46.6)
**Quality of care parameters** ^1^				
Hospital LOS (days)	805	3 [2, 4]	616	3 [2, 4]
Time to see a physician (hours)	775	2.3 [1.0, 4.0]	592	2.0 [0.5, 4.0]
**Clinical parameters at admission** ^1^				
Temperature (°C)	792	38 [37.2, 39.0]	604	38.0 [37.1, 38.9]
SpO_2_%	795	98 [95, 99]	607	97 [94, 99]
Heart rate (beats/min)	797	166 [152, 181]	609	166 [152, 182]
Lactate (mmol/L)	777	3.0 [2.0, 7.2]	597	3.4 [2.1, 8.7]
Glucose (mmol/L)	801	7.1 [5.7, 8.4]	614	7.2 [5.7, 8.7]
Nasal flaring	803	299 (37.1)	614	299 (48.5)
Subcostal retractions	804	276 (34.3)	615	276 (44.8)
Intercostal retractions	802	293 (36.4)	613	287 (46.6)
Convulsions	800	148 (18.4)	533	148 (24.0)
Coma (BCS <3)	794	70 (8.7)	608	70 (11.4)
Unable to drink/feed	799	242 (30.1)	533	242 (39.3)
**Coinfection** ^1^				
Malaria	730	340 (42.2)	544	237 (38.5)
HIV	805	22 (2.7)	616	17 (2.8)

^1^Data are presented as median [IQR] or frequency (percent) as appropriate.

BCS, Blantyre Coma Scale; IMCI, Integrated Management of Childhood Illness; IQR, interquartile range; LOS, length of stay; SpO_2_, oxygen saturation.

### Markers of immune and endothelial activation risk stratify children with IMCI pneumonia and severe pneumonia

**Fig 1:**
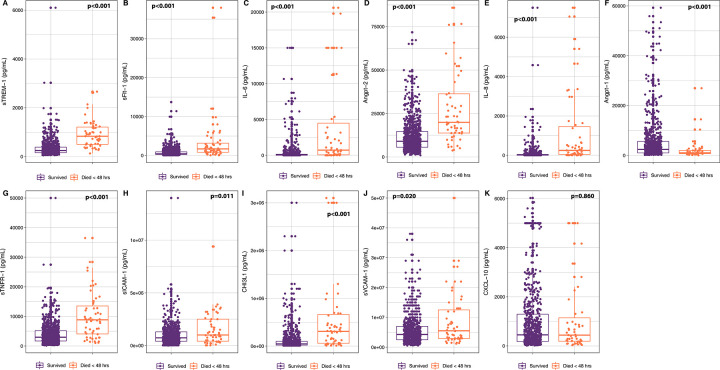
Plasma concentrations of immune and endothelial activation markers in children with IMCI pneumonia who died within 48 hours of hospital admission compared with those who survived. Plasma concentration of (a) sTREM-1 (*P* < 0.001), (b) sFlt-1 (*P* < 0.001), (c) IL-6 (*P* < 0.001), (d) Angpt-2 (*P* < 0.001), (e) IL-8 (*P* < 0.001), (f) Angpt-1 (*P* < 0.001), (g) sTNFR-1 (*P* < 0.001), (h) sICAM-1 (*P* = 0.011), (i) CHI3L1 (*P* < 0.001), (j) sVCAM-1 (*P* < 0.020), (k) CXCL-10/IP-10 (*P* = 0.860). Angpt-1, angiopoietin-1; Angpt-2, angiopoietin-2; CHI3L1, chitinase-3-like-1 protein; IL-6, interleukin-6; IL-8, interleukin-8; IMCI, Integrated Management of Childhood Illness; IP10/CXCL-10, interferon-gamma-inducible protein-10/c motif chemokine 10; sFlt-1, soluble fms-like tyrosine kinase-1; sICAM-1, soluble intracellular adhesions molecule-1; sTNFR-1, soluble tumor necrosis factor receptor-1; sTREM-1, soluble triggering receptor expressed on myeloid cells-1; sVCAM-1, soluble vascular cell adhesion molecule-1.

Plasma concentrations of all circulating markers are presented in S1 Table. Among children with pneumonia (*n* = 805), 85.9% of deaths (55 of 64) occurred within the first 48 hours of hospital admission. Plasma concentrations of immune and endothelial activation markers at presentation of children with IMCI pneumonia who subsequently died within 48 hours of hospital admission compared with those who survived is presented in Fig 1. The association of each marker with 48-hour mortality (AUROCs) in children with IMCI pneumonia is presented in Table 2. Among all children with pneumonia, the best prognostic marker of 48-hour mortality was sTREM-1, with an AUROC of 0.885 (95% CI 0.841 to 0.928). sTREM-1 had a stronger association with 48-hour mortality than the other circulating markers (Table 2). The addition of the second most discriminative marker (IL-8) to the model did not improve the model that included only sTREM-1 (AUROC = 0.885, 95% CI 0.835 to 0.936, *P* = 0.93).

**Table 2:** AUROC for the outcome of 48-hour mortality for single immune and endothelial activation marker models.

	IMCI pneumonia (*n* = 805)		Severe pneumonia (*n* = 616)	
Biomarker	AUROC (95% CI)^1^	*P* value^2^	AUROC (95% CI)^1^	*P* value^2^
sTREM-1	0.885 (0.841, 0.928)	n/a	0.870 (0.824, 0.916)	n/a
IL-8	0.794 (0.719, 0.869)	0.03	0.780 (0.703, 0.856)	0.04
sFlt-1	0.794 (0.722, 0.865)	0.01	0.775 (0.702, 0.849)	0.01
IL-6	0.793 (0.659, 0.828)	0.002	0.734 (0.649, 0.819)	0.004
Angpt-2	0.783 (0.714, 0.852)	0.004	0.763 (0.691, 0.834)	0.004
CHI3L1	0.772 (0.699, 0.845)	0.003	0.763 (0.688, 0.837)	0.007
sTNFR1	0.738 (0.719, 0.869)	<0.001	0.727 (0.643, 0.811)	<0.001
sICAM-1	0.603 (0.511, 0.694)	<0.001	0.593 (0.501, 0.684)	<0.001
sVCAM-1	0.594 (0.510, 0.678)	<0.001	0.590 (0.506, 0.675)	<0.001
IP-10/CXCL-10	0.493 (0.410, 0.575)	<0.001	0.495 (0.413, 0.578)	<0.001
Angpt-1	0.317 (0.250, 0.384)	<0.001	0.338 (0.269, 0.406)	<0.001

^1^Single variable logistic regression performed on log(e)-transformed biomarker values ranked in descending AUROC c-statistic values.

^2^*P* values represent Bonferroni-corrected AUROC model comparisons relative to top biomarker model.

AUROC, area under receiver operating characteristic curve; Angpt-1, angiopoietin-1; Angpt-2, angiopoietin-2; CHI3L1, chitinase-3-like-1 protein; CI, confidence interval; IL-6, interleukin-6; IL-8, interleukin-8; IMCI, integrated management of childhood illness; IP10/CXCL-10, interferon-gamma-inducible protein-10/c motif chemokine 10; sFlt-1, soluble fms-like tyrosine kinase-1; sICAM-1, soluble intracellular adhesions molecule-1; sTNFR-1, soluble tumor necrosis factor receptor-1; sTREM-1, soluble triggering receptor expressed on myeloid cells-1; sVCAM-1, soluble vascular cell adhesion molecule-1.

Among children with severe pneumonia (*n* = 616), sTREM-1 also showed the strongest association with 48-hour mortality (AUROC = 0.870, 95% CI 0.824 to 0.916) (Table 2). The addition of IL-8, the next most discriminating marker in children with severe pneumonia, did not improve the model relative to a model that included only sTREM-1 (AUROC = 0.872, 95% CI 0.819 to 0.924, *P* = 0.88). Plasma concentrations of each marker in children with severe pneumonia who subsequently died within 48 hours of hospital admission compared with those who survived is presented in S2 Fig.

The association of each marker with all in-hospital mortality in children with IMCI pneumonia and IMCI severe pneumonia is presented in S2 Table, with similar AUROCs as for 48-hour mortality.

### sTREM-1 is more strongly associated with 48-hour mortality than common clinical markers of pneumonia disease severity

**Fig 2:**
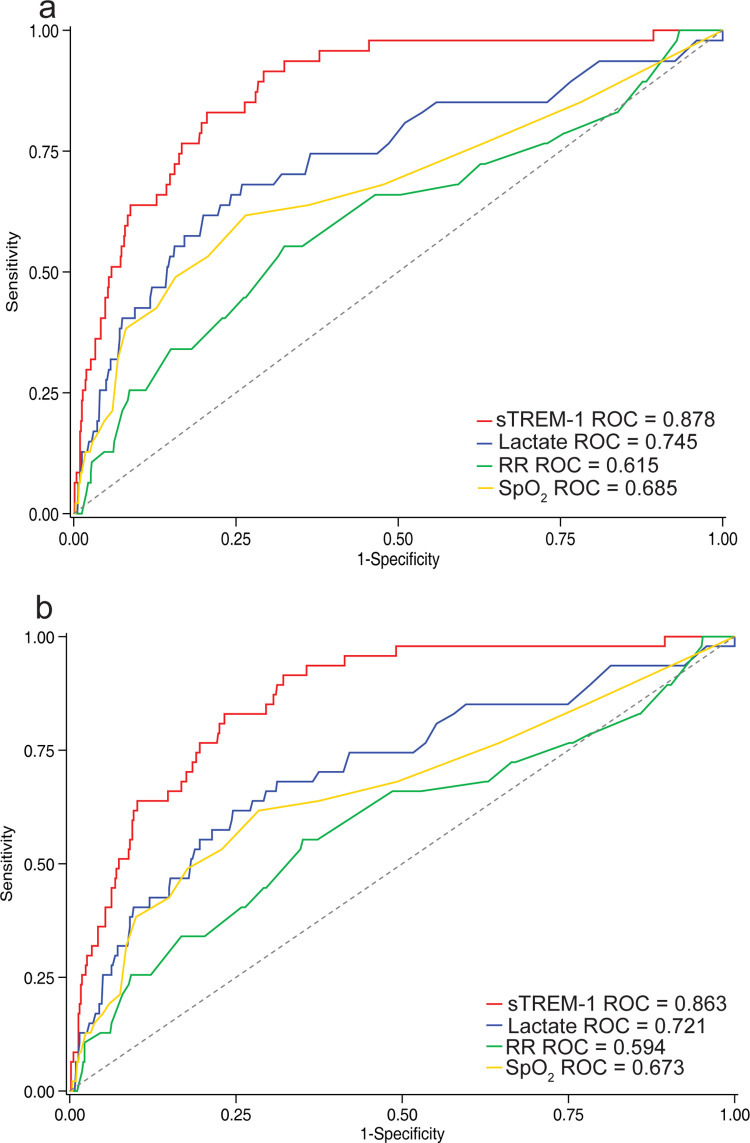
ROCs for predicting 48-hour in-hospital mortality using sTREM-1 versus common clinical parameters. (a) sTREM-1 (AUROC 0.878 95% CI 0.832–0.924) compared with lactate (AUROC 0.745, 95% CI 0.664–0.826, *P* < 0.001), RR (AUROC 0.615, 95% CI 0.528–0.702, *P* < 0.001), and oxygen saturation by pulse oximetry (SpO_2_) (AUROC 0.685, 95% CI 0.594–0.776, *P* = 0.002) in cases of IMCI pneumonia and (b) sTREM-1 (AUROC 0.863, 95% CI 0.814–0.911) compared with lactate (AUROC 0.721, 95% CI 0.638–0.803, *P* < 0.001), RR (AUROC 0.594, 95% CI 0.505–0.684, *P* < 0.001), and SpO_2_ (AUROC 0.673, 95% CI 0.582–0.764, *P* = 0.002) in cases of severe pneumonia. AUROC, area under receiver operating characteristic curve; ROC, receiver operating characteristic; RR, respiratory rate; SpO_2_, oxygen saturation; sTREM-1, soluble triggering receptor expressed on myeloid cells-1.

We examined the prognostic accuracy of sTREM-1 for 48-hour mortality relative to clinical parameters commonly used to ascertain pneumonia disease severity (lactate, respiratory rate, and oxygen saturation). sTREM-1 (AUROC = 0.878, 95% CI 0.832 to 0.924) was significantly better than lactate (AUROC = 0.745, 95% CI 0.664 to 0.826, *P* < 0.001), respiratory rate (AUROC = 0.615, 95% CI 0.528 to 0.702, *P* < 0.001), and SpO_2_ (AUROC = 0.685, 95% CI 0.594 to 0.776, *P* = 0.002) (Fig 2A and S3 Table). sTREM-1 was also stronger than the combination of respiratory rate and SpO_2_ (AUROC = 0.725, 95% CI 0.647 to 0.804, *P* < 0.001) in identifying children at risk of death within 48 hours of hospital admission.

Among children with severe pneumonia, sTREM-1 also showed a stronger association than lactate (AUROC = 0.721, 95% CI 0.638 to 0.803, *P* < 0.001), respiratory rate (AUROC = 0.594, 95% CI 0.505 to 0.684, *P* < 0.001), SpO_2_ (AUROC = 0.673, 95% CI 0.582 to 0.764, *P* = 0.002), as well as the combination of respiratory rate and SpO_2_ (AUROC = 0.706, 95% CI 0.626 to 0.786, *P* < 0.001) with 48-hour mortality (Fig 2B and S3 Table).

sTREM-1 was also significantly better than lactate, respiratory rate, and SpO_2_ in identifying all in-hospital mortality in children with IMCI pneumonia and severe pneumonia (S3 Fig and S3 Table).

### sTREM-1 is more strongly associated with 48-hour mortality than nonspecific circulating markers of inflammation

**Fig 3:**
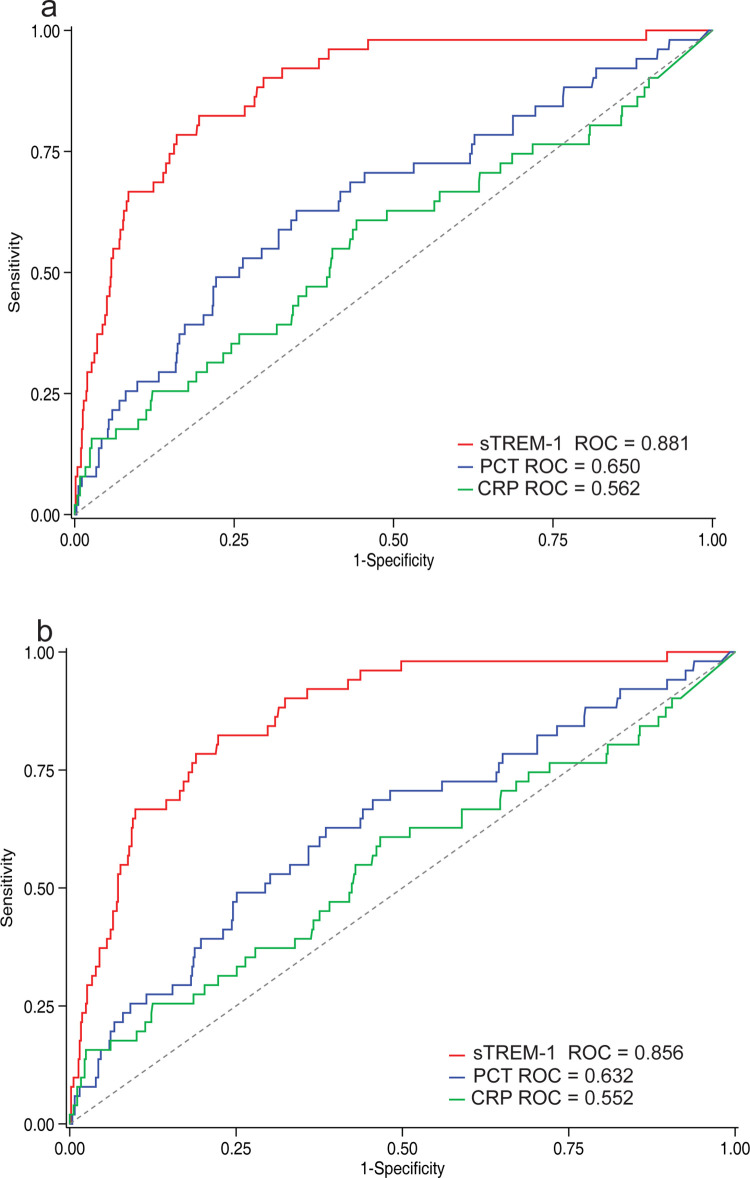
ROCs for predicting 48-hour in-hospital mortality using sTREM-1 versus nonspecific markers of inflammation. (a) sTREM-1 (AUROC 0.881, 95% CI 0.835–0.927) compared with PCT (AUROC 0.650, 95% CI 0.566–0.734, *P* < 0.001) and CRP (AUROC 0.562, 95% CI 0.472–0.653, *P* < 0.001) in cases of IMCI pneumonia and (b) sTREM-1 (AUROC 0.856, 95% CI 0.817–0.914) compared with PCT (AUROC 0.632, 95% CI 0.547–0.716, *P* < 0.001) and CRP (AUROC 0.552, 95% CI 0.461–0.644, *P* < 0.001) in cases of severe pneumonia. AUROC, area under receiver operating characteristic curve; CRP, c-reactive protein; ROC, receiver operating characteristic curve; PCT, procalcitonin; sTREM-1, soluble triggering receptor expressed on myeloid cells-1.

The association of sTREM-1 with 48-hour mortality was compared to the reference acute phase plasma markers PCT and CRP. sTREM-1 was significantly better than PCT (AUROC = 0.650, 95% CI 0.566 to 0.734, *P* < 0.001) and CRP (AUROC = 0.562, 95% CI 0.472 to 0.653, *P* < 0.001) in identifying children at risk of 48-hour death (Fig 3A and S3 Table).

Among children with severe pneumonia, the performance of sTREM-1 was also significantly better than that of PCT (AUROC = 0.632, 95% CI 0.547 to 0.716, *P* < 0.001) and CRP (AUROC = 0.552, 95% CI 0.461 to 0.644, *P* < 0.001) in identifying children at risk of 48-hour mortality (Fig 3B and S3 Table).

sTREM-1 was also significantly better than PCT and CRP for the outcome of all in-hospital mortality for children with IMCI pneumonia and severe pneumonia (S4 Fig and S3 Table).

## Discussion

Accurate triage tools to enable the early recognition of children with pneumonia at risk of progression to severe and fatal disease are lacking. This barrier leads to increased mortality among children with pneumonia and paradoxically results in unnecessary hospital admission as well as over-administration of antibiotics in children with mild and self-limited pneumonia [6]. In this prospective cohort study of children with an IMCI-defined diagnosis of pneumonia, sTREM-1 quantified at hospital presentation was significantly better than current risk-stratification strategies for 48-hour mortality including lactate, respiratory rate, oxygen saturation, as well as the acute phase markers PCT and CRP.

Early identification of children with pneumonia at risk of death can improve triage and survival. However, it is unclear how current strategies perform in recognizing these high-risk children at clinical presentation relative to circulating markers related to the pathobiology of severe infection [24,25]. Our results indicate that respiratory rate, oxygen saturation, as well as a combination of these 2 parameters measured at hospital presentation are relatively poor indicators of risk of 48-hour mortality compared to circulating protein markers of immune activation (e.g., sTREM-1). Although elevated plasma lactate in children can be indicative of disease severity [26], in this cohort, plasma lactate was also inferior to sTREM-1 in identifying children with pneumonia at risk of death.

PCT and CRP are becoming more widely used in the management of febrile pediatric syndromes and in particular pneumonia [8]. In this study, in head-to-head comparisons, both PCT and CRP were inferior to sTREM-1 in predicting mortality in children with pneumonia. These results suggest that currently used clinical tools have limited prognostic utility in identifying children at risk of fatal outcome and may be insufficient to achieve the global goal of reducing pneumonia deaths [1].

Endothelial and immune pathways regulate the host response to infection and their dysregulation contributes to disease progression and fatal outcome [12–14,27–29]. There is a growing body of evidence that measuring specific mediators of host response to acute infection (e.g., sTREM-1, Angpt-2) at clinical presentation can predict impending critical illness since they are independent and quantitative predictors of disease severity and outcome [12–14,21,27–29]. The plasma markers evaluated in this study were selected based on their role in the regulation of the host immune and endothelial response to infection.

sTREM-1 showed the strongest association with 48-hour mortality in children with pneumonia and severe pneumonia. sTREM-1 is a soluble form of a cell-surface receptor expressed on myeloid and lymphoid cells [30]. Activation of the cell-surface and soluble forms of TREM-1 results in amplification of neutrophil and monocyte responses [30], inducing NF-κB activation and the expression of inflammatory genes such as IL-6, IL-8, and TNF [31,32] as well as anti-apoptotic pathways [32–34]. In septic patients, monocyte apoptosis is inversely correlated with the expression of membrane-bound TREM-1 [32]. Excessive TREM-1 cleavage could contribute to immunosuppression and high circulating levels of soluble TREM-1 may be indicative of immune cell apoptosis. The role of sTREM-1 in the host innate immune response, as well as its performance in this study, suggest that sTREM-1 may be a reliable prognostic marker to identify children with pneumonia at risk of a poor outcome. Circulating markers with a pathobiological link to bacterial pneumonia, such as sTREM-1 [35], may guide triage and decisions about initiating, continuing, or stopping antimicrobial therapy in alignment with current pediatric treatment guidelines [36]. However, additional prospective studies measuring sTREM-1 in real time at the point of care will be required to assess its potential role in triage and clinical decision making.

Currently, sTREM-1 can be measured in multiple formats including an approximately 1-hour near-patient automated format that requires only 10 uL of plasma [22]. The results of this hospital-based study cannot be generalized into community-based settings; however, it is worth noting that to assist in early community triage of children with pneumonia in low-resource settings, sTREM-1 detection could be incorporated into a rapid and inexpensive platform, such as a point-of-care, lateral flow test. In low-resource settings, more than half of all children who die succumb to illness in rural communities without engaging the formal healthcare system [37]. This is often due to barriers to receiving hospital-based care, including the cost and distance to bring the child to a formal healthcare setting. At the community level, a rapid triage tool based on sTREM-1 could identify children with a respiratory tract infection who require referral to a hospital for immediate supportive care and antibiotic therapy. In contrast, identification of children with nonsevere pneumonia who may not require antibiotic therapy [6] could contribute to a reduction in inappropriate referrals and misuse of antibiotics, thereby reducing misallocation of health resources and decreasing antimicrobial resistance [3].

This study benefits from a prospective design, a large cohort, detailed clinical data to facilitate an IMCI-defined diagnosis of pneumonia, mortality outcomes, and a large number of host circulating markers quantified simultaneously to ensure reliable comparison. However, there are limitations. First, it was a secondary analysis in a subset of children included in a large prospective cohort study powered to assess the primary outcome of mortality in children with a febrile illness [21]. However, IMCI-defined pneumonia was a prespecified secondary analysis of the larger prospective cohort study. The goal of this secondary analysis was to compare specific, biologically relevant biomarkers to nonspecific markers of disease, to provide a rationale for developing a future point-of-care test that may be more informative and clinically relevant relative to the status quo, rather than a goal of establishing complex, multivariable prediction models. External validation will be required to verify whether sTREM-1 can predict outcome in other cohorts of children with pneumonia. Of note, the utility of sTREM-1 measurement at the time of hospital presentation to risk-stratify patients with respiratory tract infections has recently been demonstrated in adults in low-resource settings and in patients with Coronavirus Disease 2019 (COVID-19) pneumonia [38,39]. Second, the analysis only included children with complete follow up to hospital discharge and excluded children who were transferred to another hospital or who were lost to follow up, which may have contributed to prediction error. Loss to follow up is common in prospective studies in resource-limited settings, and other studies report that missing outcome values for patients lost to follow up results in an underestimation of mortality [40]. Third, radiographic imaging was not available in this study environment and as such, the diagnosis of pneumonia based only on IMCI criteria may have resulted in diagnostic misclassification. Future studies should include chest X-ray imaging to provide a more accurate diagnosis of pneumonia. However, given the limitations of chest X-ray in the diagnosis of pneumonia, including high inter-observer variability most notably in infants, tools with improved performance that are more suited to resource-constrained settings are urgently needed [41].

In conclusion, commonly used tools to assess disease severity were relatively poor predictors of pneumonia-associated mortality. sTREM-1, a circulating marker of the host immune response to infection, was more strongly associated with mortality than common clinical markers, lactate, PCT, and CRP. Measuring a host marker of immune response, such as sTREM-1, at clinical presentation may improve early triage and outcome of children with pneumonia.

## Supporting information

S1 Checklist The REMARK Checklist. (DOCX)

S1 Protocol Project proposal for Mortality and Morbidity study in children hospitalized for acute febrile illness in Uganda (Version 4, 2011). (PDF)

S1 Fig Flow chart of children included in the analysis by IMCI pneumonia and severe pneumonia. (DOCX)

S2 Fig Plasma concentrations of immune and endothelial activation markers in children with severe pneumonia who died in hospital compared with those who survived. (DOCX)

S3 Fig ROCs for predicting all in-hospital mortality using sTREM-1 versus common clinical parameters. (DOCX)

S4 Fig ROCs for predicting all in-hospital mortality using sTREM-1 versus nonspecific markers of inflammation. (DOCX)

S1 Table Plasma severity marker concentrations at hospital presentation. (DOCX)

S2 Table Area under receiver operating characteristics curve (AUROC) for the outcome of in-hospital mortality for single immune and endothelial activation marker models. (DOCX)

S3 Table Area under receiver operating characteristics (AUROC) for the outcome of 48-hour and in-hospital mortality for sTREM-1, respiratory rate, pulse oximetry, lactate, PCT, and CRP. (DOCX)

## Abbreviations

Angpt-1: angiopoietin-1
Angpt-2: angiopoietin-2
AUROC: area under the receiver operating characteristic curve
BCS: Blantyre Coma Scale
CHI3L1: chitinase-3-like-1 protein
COVID-19: Coronavirus Disease 2019
CRP: C-reactive protein
IL-6: interleukin-6
IL-8: interleukin-8
IMCI: Integrated Management of Childhood Illness
IP-10/CXCL-10: interferon-gamma-inducible protein-10/c motif chemokine 10
IQR: interquartile range
PCT: procalcitonin
RR: respiratory rate
sFlt-1: soluble fms-like tyrosine kinase-1
sICAM-1: soluble intracellular adhesions molecule-1
sTNFR-1: soluble tumor necrosis factor receptor-1
sTREM-1: soluble triggering receptor expressed on myeloid cells-1
sVCAM-1: soluble vascular cell adhesion molecule
WHO: World Health Organization

## References

[1] Every Breath Counts SP. Every Breath Counts Campaign [cited 2019]. Available from: https://stoppneumonia.org/research/. Last accessed: April 21, 2022.

[2] Liu L, Oza S, Hogan D, Perin J, Rudan I, Lawn JE, et al. Global, regional, and national causes of child mortality in 2000–13, with projections to inform post-2015 priorities: an updated systematic analysis. Lancet. 2015;385(9966):430–40. Epub 2014/10/05. doi: 10.1016/S0140-6736(14)61698-6 . 25280870

[3] Launay E, Le Guen CG. Antibiotic prescription in paediatric emergency departments: fear and reason. Lancet Infect Dis. 2019;19(4):341–2. doi: 10.1016/S1473-3099(18)30727-8 . 30827809

[4] Rudan I, Boschi-Pinto C, Biloglav Z, Mulholland K, Campbell H. Epidemiology and etiology of childhood pneumonia. Bull World Health Organ. 2008;86(5):408–16. Epub 2008/06/12. doi: 10.2471/blt.07.048769 ; PubMed Central PMCID: PMC2647437. 18545744 PMC2647437

[5] Benet T, Picot VS, Awasthi S, Pandey N, Bavdekar A, Kawade A, et al. Severity of Pneumonia in under 5-year-old children from developing countries: A multicenter, prospective, observational study. Am J Trop Med Hyg. 2017;97(1):68–76. doi: 10.4269/ajtmh.16-0733 ; PubMed Central PMCID: PMC5508893. 28719310 PMC5508893

[6] D’Acremont V, Kilowoko M, Kyungu E, Philipina S, Sangu W, Kahama-Maro J, et al. Beyond malaria—causes of fever in outpatient Tanzanian children. N Engl J Med. 2014;370(9):809–17. Epub 2014/02/28. doi: 10.1056/NEJMoa1214482 . 24571753

[7] Rambaud-Althaus C, Althaus F, Genton B, D’Acremont V. Clinical features for diagnosis of pneumonia in children younger than 5 years: a systematic review and meta-analysis.Lancet Infect Dis. 2015;15(4):439–50. Epub 2015/03/15. doi: 10.1016/S1473-3099(15)70017-4 . 25769269

[8] van Griensven J, Cnops L, De Weggheleire A, Declercq S, Bottieau E. Point-of-care biomarkers to guide antibiotic prescription for acute febrile illness in sub-Saharan Africa: Promises and caveats. Open Forum Infect Dis. 2020;7(8):ofaa260. Epub 2020/08/21. doi: 10.1093/ofid/ofaa260 ; PubMed Central PMCID: PMC7423291. 32818139 PMC7423291

[9] WHO. Handbook: IMCI integrated management of childhood illness. Geneva, Switzerland: 2005.

[10] Ginsburg AS, Lenahan JL, Izadnegahdar R, Ansermino JM. A systematic review of tools to measure respiratory rate in order to identify childhood pneumonia. Am J Respir Crit Care Med. 2018;197(9):1116–27. doi: 10.1164/rccm.201711-2233CI . 29474107

[11] Ginsburg AS, Mvalo T, Nkwopara E, McCollum ED, Ndamala CB, Schmicker R, et al. Placebo vs amoxicillin for nonsevere fast-breathing pneumonia in Malawian children aged 2 to 59 months: A double-blind, randomized clinical noninferiority trial. JAMA Pediatr. 2019;173(1):21–8. Epub 2018/11/13. doi: 10.1001/jamapediatrics.2018.3407 ; PubMed Central PMCID: PMC6583426. 30419120 PMC6583426

[12] Xing K, Murthy S, Liles WC, Singh JM. Clinical utility of biomarkers of endothelial activation in sepsis—a systematic review. Crit Care. 2012;16(1):R7. Epub 2012/01/18. doi: 10.1186/cc11145 ; PubMed Central PMCID: PMC3396237. 22248019 PMC3396237

[13] Leligdowicz A, Richard-Greenblatt M, Wright J, Crowley VM, Kain KC. Endothelial Activation: The Ang/Tie Axis in Sepsis. Front Immunol. 2018;9:838. Epub 2018/05/10. doi: 10.3389/fimmu.2018.00838 ; PubMed Central PMCID: PMC5928262. 29740443 PMC5928262

[14] Ghosh CC, David S, Zhang R, Berghelli A, Milam K, Higgins SJ, et al. Gene control of tyrosine kinase TIE2 and vascular manifestations of infections. Proc Natl Acad Sci U S A. 2016;113(9):2472–7. Epub 2016/02/18. doi: 10.1073/pnas.1519467113 ; PubMed Central PMCID: PMC4780619. 26884170 PMC4780619

[15] Higgins SJ, Purcell LA, Silver KL, Tran V, Crowley V, Hawkes M, et al. Dysregulation of angiopoietin-1 plays a mechanistic role in the pathogenesis of cerebral malaria. Sci Transl Med. 2016;8(358):358ra128. Epub 2016/09/30. doi: 10.1126/scitranslmed.aaf6812 . 27683553 PMC6450386

[16] Hendrickson CM, Matthay MA. Endothelial biomarkers in human sepsis: pathogenesis and prognosis for ARDS. Pulm Circ. 2018;8(2):2045894018769876. Epub 2018/03/27. doi: 10.1177/2045894018769876 ; PubMed Central PMCID: PMC5912282. 29575977 PMC5912282

[17] Valim C, Ahmad R, Lanaspa M, Tan Y, Acacio S, Gillette MA, et al. Responses to bacteria, virus, and malaria distinguish the etiology of pediatric clinical pneumonia. Am J Respir Crit Care Med. 2016;193(4):448–59. Epub 2015/10/16. doi: 10.1164/rccm.201506-1100OC ; PubMed Central PMCID: PMC5440057. 26469764 PMC5440057

[18] Erdman LK, Petes C, Lu Z, Dhabangi A, Musoke C, Cserti-Gazdewich CM, et al. Chitinase 3-like 1 is induced by Plasmodium falciparum malaria and predicts outcome of cerebral malaria and severe malarial anaemia in a case-control study of African children. Malar J. 2014;13:279. Epub 2014/07/23. doi: 10.1186/1475-2875-13-279 ; PubMed Central PMCID: PMC4114103. 25047113 PMC4114103

[19] Mikacenic C, Hahn WO, Price BL, Harju-Baker S, Katz R, Kain KC, et al. Biomarkers of endothelial activation are associated with poor outcome in critical illness. PLoS ONE. 2015;10(10):e0141251. Epub 2015/10/23. doi: 10.1371/journal.pone.0141251 ; PubMed Central PMCID: PMC4619633. 26492036 PMC4619633

[20] Lee WL, Liles WC. Endothelial activation, dysfunction and permeability during severe infections. Curr Opin Hematol. 2011;18(3):191–6. Epub 2011/03/23. doi: 10.1097/MOH.0b013e328345a3d1 . 21423012

[21] Leligdowicz A, Conroy AL, Hawkes M, Richard-Greenblatt M, Zhong K, Opoka RO, et al. Risk-stratification of febrile African children at risk of sepsis using sTREM-1 as basis for a rapid triage test. Nat Commun. 2021;12(1):6832. Epub 2021/11/27. doi: 10.1038/s41467-021-27215-6 ; PubMed Central PMCID: PMC8617180. 34824252 PMC8617180

[22] Leligdowicz A, Conroy AL, Hawkes M, Zhong K, Lebovic G, Matthay MA, et al. Validation of two multiplex platforms to quantify circulating markers of inflammation and endothelial injury in severe infection. PLoS ONE. 2017;12(4):e0175130. Epub 2017/04/19. doi: 10.1371/journal.pone.0175130 ; PubMed Central PMCID: PMC5395141. 28419100 PMC5395141

[23] Conroy AL, Hawkes M, Hayford K, Namasopo S, Opoka RO, John CC, et al. Prospective validation of pediatric disease severity scores to predict mortality in Ugandan children presenting with malaria and non-malaria febrile illness. Crit Care. 2015;19:47. Epub 2015/04/17. doi: 10.1186/s13054-015-0773-4 ; PubMed Central PMCID: PMC4339236. 25879892 PMC4339236

[24] Molyneux E, Ahmad S, Robertson A. Improved triage and emergency care for children reduces inpatient mortality in a resource-constrained setting. Bull World Health Organ. 2006;84(4):314–9. Epub 2006/04/22. doi: 10.2471/blt.04.019505 ; PubMed Central PMCID: PMC2627321. 16628305 PMC2627321

[25] Nolan T, Angos P, Cunha AJ, Muhe L, Qazi S, Simoes EA, et al. Quality of hospital care for seriously ill children in less-developed countries. Lancet. 2001;357(9250):106–10. Epub 2001/02/24. doi: 10.1016/S0140-6736(00)03542-X . 11197397

[26] Standage SW, Wong HR. Biomarkers for pediatric sepsis and septic shock. Expert Rev Anti Infect Ther. 2011;9(1):71–9. Epub 2010/12/22. doi: 10.1586/eri.10.154 ; PubMed Central PMCID: PMC3033193. 21171879 PMC3033193

[27] Page AV, Liles WC. Biomarkers of endothelial activation/dysfunction in infectious diseases. Virulence. 2013;4(6):507–16. doi: 10.4161/viru.24530 ; PubMed Central PMCID: PMC5359744. 23669075 PMC5359744

[28] Kim H, Higgins S, Liles WC, Kain KC. Endothelial activation and dysregulation in malaria: a potential target for novel therapeutics. Curr Opin Hematol. 2011;18(3):177–85. doi: 10.1097/MOH.0b013e328345a4cf . 21423010

[29] Page AV, Kotb M, McGeer A, Low DE, Kain KC, Liles WC. Systemic dysregulation of angiopoietin-1/2 in streptococcal toxic shock syndrome. Clin Infect Dis. 2011;52(8):e157–61. doi: 10.1093/cid/cir125 . 21460306

[30] Klesney-Tait J, Turnbull IR, Colonna M. The TREM receptor family and signal integration. Nat Immunol. 2006;7(12):1266–73. Epub 2006/11/18. doi: 10.1038/ni1411 . 17110943

[31] Roe K, Gibot S, Verma S. Triggering receptor expressed on myeloid cells-1 (TREM-1): A new player in antiviral immunity? Front Microbiol. 2014;5:627. doi: 10.3389/fmicb.2014.00627 ; PubMed Central PMCID: PMC4244588. 25505454 PMC4244588

[32] Cai M, Chen Q, Chen C, Liu X, Hou J, Zeng C, et al. Activation of triggering receptor expressed on myeloid cells-1 protects monocyte from apoptosis through regulation of myeloid cell leukemia-1. Anesthesiology. 2013;118(5):1140–9. doi: 10.1097/ALN.0b013e31828744a5 . 23364598

[33] Yuan Z, Syed MA, Panchal D, Joo M, Colonna M, Brantly M, et al. Triggering receptor expressed on myeloid cells 1 (TREM-1)-mediated Bcl-2 induction prolongs macrophage survival. J Biol Chem. 2014;289(21):15118–29. doi: 10.1074/jbc.M113.536490 ; PubMed Central PMCID: PMC4031561. 24711453 PMC4031561

[34] Radsak MP, Salih HR, Rammensee HG, Schild H. Triggering receptor expressed on myeloid cells-1 in neutrophil inflammatory responses: differential regulation of activation and survival. J Immunol. 2004;172(8):4956–63. doi: 10.4049/jimmunol.172.8.4956 . 15067076

[35] Sibila O, Restrepo MI. Biomarkers in community-acquired pneumonia: still searching for the one. Eur Respir J. 2019;53(2). Epub 2019/03/02. doi: 10.1183/13993003.02469–2018 . 30819808

[36] Weiss SL, Peters MJ, Alhazzani W, Agus MSD, Flori HR, Inwald DP, et al. Surviving sepsis campaign international guidelines for the management of septic shock and sepsis-associated organ dysfunction in children. Pediatr Crit Care Med. 2020;21(2):e52–e106. Epub 2020/02/08. doi: 10.1097/PCC.0000000000002198 . 32032273

[37] Rutherford ME, Dockerty JD, Jasseh M, Howie SR, Herbison P, Jeffries DJ, et al. Access to health care and mortality of children under 5 years of age in the Gambia: A case-control study. Bull World Health Organ. 2009;87(3):216–24. Epub 2009/04/21. doi: 10.2471/blt.08.052175 ; PubMed Central PMCID: PMC2654650. 19377718 PMC2654650

[38] Van Singer M, Brahier T, Ngai M, Wright J, Weckman AM, Erice C, et al. COVID-19 risk stratification algorithms based on sTREM-1 and IL-6 in emergency department. J Allergy Clin Immunol. 2020. Epub 2020/10/13. doi: 10.1016/j.jaci.2020.10.001 ; PubMed Central PMCID: PMC7546666. 33045281 PMC7546666

[39] Richard-Greenblatt M, Boillat-Blanco N, Zhong K, Mbarack Z, Samaka J, Mlaganile T, et al. Prognostic accuracy of soluble triggering receptor expressed on myeloid cells (sTREM-1)-based algorithms in febrile adults presenting to Tanzanian outpatient clinics. Clin Infect Dis. 2020;70(7):1304–12. Epub 2019/05/19. doi: 10.1093/cid/ciz419 . 31102510

[40] Taylor T, Olola C, Valim C, Agbenyega T, Kremsner P, Krishna S, et al. Standardized data collection for multi-center clinical studies of severe malaria in African children: establishing the SMAC network. Trans R Soc Trop Med Hyg. 2006;100(7):615–22. Epub 2006/03/23. doi: 10.1016/j.trstmh.2005.09.021 ; PubMed Central PMCID: PMC1459261. 16551469 PMC1459261

[41] Davies HD, Wang EE, Manson D, Babyn P, Shuckett B. Reliability of the chest radiograph in the diagnosis of lower respiratory infections in young children. Pediatr Infect Dis J. 1996;15(7):600–4. doi: 10.1097/00006454-199607000-00008 8823854

