# Supplementary material for: Immune and endothelial activation markers and risk stratification of childhood pneumonia in Uganda: A secondary analysis of a prospective cohort study
Source: PLoS Med. 2022 Jul 13;19(7):e1004057. doi: 10.1371/journal.pmed.1004057 (PMC9328519; doi:10.1371/journal.pmed.1004057)
Supplement: S1 Protocol — (PDF) [file pmed.1004057.s002.pdf]

Title of Project: Predictive biomarkers of mortality and morbidity in children hospitalized for acute febrile illness

Principal investigator (Uganda): Dr. Robert Opika Opoka, *MBChB, MMED, MPH*
Department of Pediatrics & Child Health, Mulago Hospital

Principal investigator (Canada): Prof. Kevin Kain, *MD, FRCPC,*
Director of Global Health, McLaughlin Center for Molecular Medicine, University of Toronto

Co-Investigators:
Dr. Michael Hawkes, *MD, FRCPC*, Institute of Medical Sciences, University of Toronto
Dr. Sophie Namasopo, *MBChB, MMED,* Jinja Regional Referral Hospital
Dr. Chandy C. John, *MD, MS*, Department of Pediatrics, University of Minnesota.
Dr. W. Conrad Liles, *MD, PhD, FRCPC*, Dept of Medicine, University of Toronto
Dr. Andrea Conroy, *PhD*, Medicine, University of Toronto
Dr. Laura Hermann, *PhD*, *MD, FRCPC*, Medicine, University of Toronto

BACKGROUND AND SIGNIFICANCE

Introduction

Approximately 7.7 million children per year die before the age of 5 years. Infectious diseases account for a large proportion of these deaths, including pneumonia (2.1 million deaths/year), malaria (1 million deaths/year), HIV/AIDS and measles [1]. Most deaths occur in resource-poor settings in Asia and Africa [2]. Large gaps remain in the case management of children presenting to African hospitals with severe febrile syndromes, and new tools to assist practitioners in triage, diagnosis and treatment decisions are a priority. We propose to investigate a panel of biomarkers of established pathways leading to critical illness for their diagnostic, predictive and prognostic utility in children presenting to hospital with severe febrile illness.

Malaria is the most common diagnosis among hospitalized children in Africa [3]. Malaria is also the leading parasitic cause of morbidity and mortality worldwide, causing an estimated 515 million clinical cases and ~1-2 million deaths annually [4]. Despite the use of highly effective anti-malarial medications, 10-30% patients with severe malaria will die [5], underscoring the need for new methods to identify patients at greatest risk in order to efficiently allocate scarce resources to those who need them most. Because the clinical signs and symptoms of malaria are non-specific, differentiating it from other causes of acute febrile illness can be difficult.

On the other hand, in an era of increasing cost of antimalarial treatment, rollout of accurate malaria rapid diagnostic tests (RDTs), and a declining burden of malaria in sub-Saharan Africa [6-9], management of febrile children without evidence of malaria is another emerging clinical problem. Management of febrile patients in Africa without evidence of malaria is now a research priority for several major international donor organizations, including the Bill and Melinda Gates Foundation. An extensive differential diagnosis confounds the clinical management of this group of patients, in whom the mortality is often even higher than malaria-positive children [10]. Our study will therefore explore the clinical utility of host biomarkers in both malaria-positive and malaria-negative patients, and their ability to distinguishing between malaria and serious bacterial infection (e.g., pneumonia, sepsis and meningitis).

Our prospective observational study will link admission levels of key host biomarkers of inflammation, angiogenesis, coagulation and endothelial stability to meaningful clinical outcomes (i.e., mortality and length of admission). This information may demonstrate the potential clinical utility of these biomarkers, individually and collectively, for patient triage, diagnosis and assignment to advanced therapeutics. Our findings may accelerate the development of tools for case management that can assist practitioners in resource-constrained African hospitals providing health care to children with severe febrile syndromes. New tools for clinical care, in turn, may save lives in resource-constrained settings by focusing efforts toward improved child survival.

Objective

The objective of this proposal is to identify clinically informative biomarkers of host defense pathways with potential utility as diagnostic and prognostic tools among children hospitalized with acute febrile illness in a resource-constrained sub-Saharan African setting.

Our specific aims are:

1. To quantify peripheral blood levels of key indicator proteins in inflammatory, angiogenic, coagulation and endothelial activation pathways, at admission, among African children hospitalized for acute febrile illness.
2. To compare biomarker levels among subgroups of hospitalized patients, including children with malaria and serious bacterial infection (pneumonia, sepsis and/or meningitis). This may offer insight into pathogenic mechanisms and engagement of specific host defense programs in common pediatric causes of mortality and disability. To apply combinatorial techniques to determine if characteristic signatures of biomarkers can differentiate between clinical syndromes and assist in diagnosis and management.
3. To examine the association between biomarker levels and length of hospital stay, as a marker of disease severity and morbidity.
4. To compare biomarker levels between survivors and non-survivors, in order to identify biomarkers that may serve as clinically informative prognostic and/or triage tools.

The working hypothesis is that a panel of biomarkers, readily measurable from a peripheral blood sample, may serve as a clinically useful instrument to distinguish between common pediatric causes of fever, predict those children at greatest need of aggressive supportive care and/or adjunctive therapies, and identify those children at greatest risk of mortality. The use of objective and quantitative tools may facilitate the triage and clinical care of febrile children admitted to hospital in the sub-Saharan African context.

BACKGROUND AND SIGNIFICANCE

Globally, nearly 7.7 million children die annually [2]. It is estimated that 63% of these deaths are avoidable with currently available interventions [11]. One major challenge in implementing effective treatment measures, particularly in resource-limited settings, is identifying those patients at greatest risk of adverse outcomes. Triage tools that would allow efficient allocation of resources to those in greatest need could streamline health care provision and potentially save lives. Furthermore, limitations in the number and training of health care providers in many areas of the world where childhood mortality is highest mean that diagnostic judgments are often rendered by minimally trained community health workers, or overburdened and time-strapped nurses and physicians. Objective and practical diagnostic aides for common, life-

threatening pediatric syndromes could assist practitioners to rapidly arrive at accurate diagnosis for improved case management. Finally, there is a need for predictive instruments which could identify patients that stand to benefit from specific novel or adjunctive therapies.

The most common causes of death in children under five years of age are pneumonia, malaria, diarrheal illness, malnutrition, measles, and HIV/AIDS [2]. Evidence-based life-saving interventions exist for these conditions, including oral and intravenous rehydration therapy, antibiotics, antimalarials and anti-retroviral medications. Moreover, scientific and technological advances are expanding the armamentarium available to combat child mortality. Yet many interventions are not reaching those at greatest need [11]. Levels of coverage with these interventions are still low in most low-income and middle-income countries and delivery of some interventions has stagnated or is even falling in several of the poorest countries [12]. In resource-constrained hospitals as in sub-Saharan Africa, the enormous need is coupled with a severe lack of resources. In this context, appropriate patient triage, risk stratification, and resource allocation are of primary importance, and tools are urgently needed to target existing or novel therapeutics to children who need them most.

Malaria is the most common diagnosis in most hospitals in Africa and consumes a large proportion of available resources [3]. Clinical diagnosis is challenging because of the low specificity of signs and symptoms of disease. Laboratory diagnosis using light microscopy is the current gold standard, but is hampered by lack of quality control, lack of quality equipment and reagents, and high laboratory workload. Lateral-flow immunochromatographic RDTs offer a practical point-of-care diagnostic alternative to microscopy. Nonetheless, limited diagnostic capacity for other infectious diseases results in frequent prescription of antimalarials to microscopy-negative and/or RDT-negative cases [3]. Overdiagnosis of malaria is thus widespread in African hospitals, resulting in the inappropriate use of antimalarial medications [3]. Additional diagnostic tools that could distinguish among the causes of febrile illness may help to limit the use of antimalarial agents, and illuminate alternative appropriate therapy, in patients without malaria.

The burden of malaria is falling in many parts of sub-Saharan Africa [6-9]. A broad array of alternative causes of undifferentiated fever, including occult bacteremia, focal bacterial infections, leptospirosis, rickettsial disease, tuberculosis, and viral infections, are thus increasing in relative importance among children hospitalized for fever. Many of these conditions are treatable with widely available antimicrobials, such that appropriate case management of malaria-negative patients will require improved diagnostic methods appropriate for field use. With the increasing promotion of parasitologic diagnosis (microscopy- or RDT-based) over syndromic diagnosis and treatment, in part driven by higher costs of first-line anti-malarial agents, the management of malaria-negative febrile patients will become more of a priority. Improved diagnostic and prognostic tools in this group of patients are therefore needed.

The signs and symptoms associated with poor outcome in severe childhood malaria are also predictive of poor outcome in febrile children without malaria [3]. Respiratory distress and altered consciousness were the strongest predictors of mortality in both microsopy-positive and microscopy-negative children with severe febrile illness in one study in Tanzania [3]. These findings emphasize the importance of host response to infectious disease, irrespective of the invading pathogen, and suggest that "agnostic" pathophysiologic assessment, irrespective of microbial etiology, may offer important prognostic information in severe febrile illness.

Severe infection due to a range of viral, bacterial and parasitic causes is characterized by engagement of common host defense pathways, including inflammation, angiogenesis, coagulation and endothelial activation. Malaria, pneumonia, sepsis, diarrheal illness, and HIV/AIDS are common infectious causes of pediatric mortality and morbidity in which activation of host defense pathways has been implicated in disease progression. Dysregulated inflammatory responses and endothelial activation are thought to be central processes in sepsis and severe malaria pathogenesis [13-15]. We hypothesize that peripheral blood biomarkers of these pathways may have clinical utility as prognostic tools. Furthermore, combinatorial approaches, using the information provided by multiple biomarkers from distinct pathobiological pathways may improve predictive ability of biomarkers [16]. We will examine key regulatory and/or indicator proteins within these pathways for their utility as indices of disease severity and outcome in Ugandan children presenting to hospital for any cause.

Inflammation
Excessive pro-inflammatory responses to infection are observed in severe infections such as malaria, pneumonia, and sepsis [17]. The acute-phase reactants C-reactive protein (CRP) and procalcitonin (PCT) are elevated in the setting of bacterial infection and malaria [18]. These markers have been used in clinical practice to distinguish between severe bacterial infection and benign causes of fever in children. Other potentially informative inflammatory markers include the 10 kDa interferon gamma-induced protein (IP-10), a chemokine elevated in fatal malaria [19], and soluble triggering receptor expressed on myeloid cells-1 (sTREM-1). We will assess these host protein among children hospitalized for any cause to determine their clinical utility.

The triggering receptor expressed on myeloid cells-1 (TREM-1) mediates pro-inflammatory responses [20]. Inhibition of TREM-1 improves outcome in murine models of sepsis [21]. Membrane density of TREM-1 on peripheral monocytes appears to be increased in patients with uncomplicated malaria cases compared to uninfected individuals [22]. Soluble TREM-1 (sTREM-1) is generated by cleavage of membrane TREM-1 upon myeloid cell activation [23], thereby serving as peripheral blood marker of leucocyte stimulation. Both membrane and sTREM-1 are increased in inflammatory pathologies in humans [24]. Given the role of TREM-1 in leucocyte activation and the association of sTREM-1 with inflammatory states, we hypothesize

that sTREM-1 will serve as a useful biomarker for the prediction of in-hospital mortality and morbidity in a cohort of African children.

Endothelial activation

Widespread endothelial activation with capillary leak leads to multi-system organ failure in severe sepsis [15] and endothelial activation in the brain vasculature may account in part for the manifestations of cerebral malaria [16]. Following activation by pro-inflammatory stimuli, soluble endothelial cell receptors are released into the circulation via ectodomain shedding or alternative splicing, such that peripheral blood levels of key regulatory proteins reflect the state of whole body endothelial activation. We will examine several critical molecules involved in the regulation of endothelial quiescence and activation.

Intercellular adhesion molecule-1 (ICAM-1) is a cell surface glycoprotein that mediates sequestration of parasitized erythrocytes in the brain microvasculature, leading to vessel occlusion [25] and blood-brain barrier dysfunction [26]. The soluble form of ICAM-1 (sICAM-1) is increased in sepsis and severe malaria [27, 28]. Likewise, the cell surface receptor *P-selectin* may be shed from the endothelium in severe disease. We hypothesize that elevated levels of sICAM-1 and/or sP-selectin may also predict adverse outcomes in hospitalized African children.

Soluble FMS-like tyrosine kinase-1 (sFlt-1) is generated by alternative splicing of VEGF receptor-1 mRNA and antagonizes the pro-inflammatory and pro-angiogenic effects of VEGF. sFlt-1 is elevated in children with severe malaria as well as sepsis patients [29]. Data from murine models of sepsis suggest that sFlt-1 may play a modulatory role [30], counteracting increased VEGF tissue expression and plasma levels, associated with cerebral malaria [31]. Similarly, *endoglin*, a component of the tumor necrosis factor-beta (TGF-β) receptor complex, participates in inflammatory signaling pathways. The soluble form of the receptor (s-endoglin) is shed from the endothelial surface into the circulation in the setting of critical illness. We hypothesize that sFlt-1 and/or s-endoglin may be clinically informative markers of disease severity in hospitalized African children.

Angiopoietins (Angs) represent a distinct family of vascular growth factors that are increasingly recognized for their role in infectious disease pathogenesis. Four molecules have been characterized, Ang-1, Ang-2, Ang-3, and Ang-4, which signal through the Tie-2 receptor on endothelial cells. Ang-1 is secreted from pericytes within the vascular intima, whereas Ang-2 is stored with von Willebrand factor in Wiebel-Palade bodies. Ang-1 and Ang-2 act antagonistically: Ang-1 promotes stability of the vascular endothelium and Ang-2 promotes permeability of the blood-tissue barrier [15, 32]. Ang-2 also functions as an autocrine regulator by sensitizing the endothelium to the effects of TNF, resulting in increased adhesion receptor expression [33]. Like other endothelial surface molecules, the Tie-2 receptor is released into the circulation in severe illness. Our group has previously described the clinical utility of peripheral blood angiopoietins and sTie-2 for prognosis in sepsis

[15] and malaria [16, 34, 35]. We hypothesize that their clinical utility may also be extended to common childhood illnesses requiring hospital admission.

Coagulation
Coagulopathy is a well-recognized manifestation of several severe infectious syndromes such as meningococcemia, sepsis and severe malaria. In addition, regulation of the coagulation cascade is intimately linked to the vascular endothelium, such that alterations in in the profile of coagulation proteins also reflect activation and/or dysfunction of the endothelium. Like Ang-2, vWF and its precursor, vWF propeptide, are stored within WP bodies and released into the circulation in response to inflammatory stimuli. In severe malaria, vWF may help tether parasitized erythrocytes to endothelial cells via platelets [36]. We plan to evaluate vWF and its precursor, vWF propeptide, as potential markers of severe disease in hospitalized African children.

Expertise of the collaborative research group

Our collaborative research group is ideally suited to conduct the research study proposed. Kevin Kain (PI) has led research teams and authored numerous publications ranging from molecular pathogenesis of malaria to randomized clinical trials of novel therapies[37-39]. The Kain group has published seminal work on biomarkers for malaria [16, 34, 35]. Robert Opoka (Ugandan PI of this study) has published numerous reports on the management of febrile children and malaria specifically in Uganda. Chandy John (Co-PI) has conducted extensive research in malaria immunology and epidemiology in Uganda and elsewhere. The combined research expertise of our collaborative team in clinical pediatric infectious diseases, malaria, field trials in resource-constrained settings, and biomarkers of disease processes makes us uniquely qualified to successfully complete this proposed research.

RESEARCH DESIGN AND METHODS

Study Design: *Prospective matched case-control study.* The explanatory (independent) variables are the quantitative biomarker levels, measured at hospital admission. The primary outcome is all-cause in-hospital mortality. The duration of hospital admission and clinical severity score will also be examined as secondary outcomes (surrogate marker of disease severity and morbidity). From this prospectively enrolled cohort, all fatal cases and a subset of age- and sex-matched non-fatal controls (1 case: 3 control ratio) will be tested for biomarker levels. Of note, both the explanatory variables, (samples for biomarker testing) and the outcomes (mortality, length of hospital stay, clinical severity score) will be assessed prospectively. Outcome assessment will be blinded to the biomarker levels, and

laboratory technicians performing the assays will be blinded to the outcome. Figure 1 presents a flowchart for this study design.

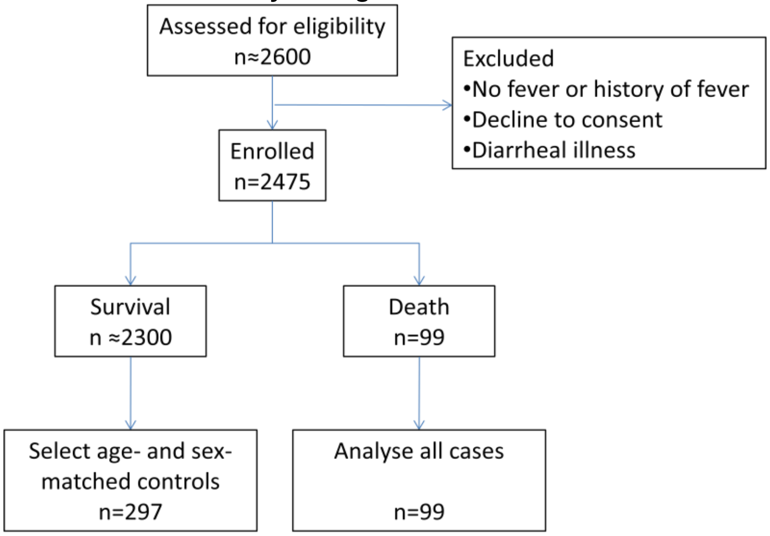

Figure 1. Flowchart for prospective enrolment, and case-control selection.

Participants

Patients age 2 months to 5 years admitted to Jinja Regional Referral hospital with a history of fever or axillary temperature > 37.5°C.

Study site

The Jinja Regional Referral Hospital serves 6 districts in mid eastern Uganda. It is also a training hospital for the school of nursing. The hospital has 2 campuses; a wing containing the children unit and larger wing for the rest of the patients as well as the administration and main laboratory. The children's unit is staffed by a clinical team that includes 5 paediatricians.

Inclusion criteria

1. Age 2 months to 5 years
2. History of fever within past 48 hours or axillary temperature >37.5°C
3. Hospital admission warranted based on clinician judgment
4. Consent to blood sampling and data collection

Exclusion criteria

1. Outside eligible age range
2. No history or objective evidence of fever
3. Diarrheal illness without other symptoms
4. Outpatient management
5. Denial of consent to participate in study

The reason for exclusion of isolated diarrheal illness is that dehydration, acidosis and electrolyte imbalances are the proximal causes of mortality, rather than activation of host defense pathways with excessive inflammation and endothelial activation. Host biomarkers are therefore not expected to predict mortality with the same accuracy in diarrhea syndromes.

Explanatory (independent) variables

The study aims to examine activation of critical illness pathways (inflammation, angiogenesis, coagulation and endothelial stability) in pediatric febrile illnesses. This will be achieved using several selected peripheral blood proteins, alone and in combination, as quantitative biomarkers of critical pathobiological pathways. These will be measured using commercially-available enzyme-linked immunosorbent assays (ELISA). Relevant biomarkers from critical illness pathways will includethe following (although additional biomarkers may be considered as information becomes available in the literature):

Biomarkers of inflammation

- soluble endoglin
- soluble FMS-like tyrosine kinase-1 (Flt-1)
- C-reactive protein
- procalcitonin
- 10 kDa interferon gamma-induced protein (IP-10)
- soluble triggering receptor expressed on myeloid cells-1 (TREM-1)

Biomarkers of coagulation

- von Willebrand Factor (vWF)
- vWF propeptide

Biomarkers of angiogenesis and endothelial stability

- Angiopoietin-1
- Angiopoietin-2
- soluble Tie-2
- soluble P-selectin
- soluble intercellular adhesion molecule-1 (ICAM-1)

Co-variates

- Age
- Gender
- HIV status

- *P. falciparum* antigenemia (malaria RDT result)
- Category of severe febrile illness (malaria, non-malarial severe febrile illness, clinically diagnosed serious bacterial infection)
- Indices of nutrition status: weight, height, mid-upper arm circumference (MUAC)
- Clinical prognostic signs: respiratory distress, altered level of consciousness, seizures, Blantyre coma score (BCS)
- Biochemical prognostic markers: lactate concentration, creatinine

Outcomes

Primary outcome measure: Inpatient mortality.

Secondary outcome measures:

- Length of hospital stay. This represents a surrogate marker of disease severity and morbidity.
- Clinical severity scores: SICK (Signs of Inflammation in Children that can Kill) and LODS (Lambaréné Organ Dysfunction Score) [40]

Several alternative clinical severity scoring systems have been published including: PRISM (Pediatric Risk of Mortality) [41], PIM (Pediatric Index of Mortality [41], sMODS (simplified Multi-Organ Dysfunction Score), PELOD (paediatric logistic organ dysfunction) [42]. These scoring systems, unlike SICK and LODS, make use of laboratory parameters to derive a total prognostic score, which are not available in our resource-limited hospital, as elsewhere in other African settings where child mortality is highest [43].

Sample size

We will enroll a total of 2475 hospitalized patients. All the expected non-survivors (n=99; cases) and 297 survivors (controls) will be further analysed for peripheral blood biomarker levels.

This sample size was calculated as follows. We used a 3:1 ratio of controls (survivors) to cases (non-survivors). We assumed a mortality rate among hospitalized patients of 4% based on surveillance data from Jinja Regional Referral Hospital (unpublished data). We took the case of Ang-2 as a prototypical biomarker, assumed a normal distribution of the data and used levels from a past study in severe malaria [44] to estimate the expected baseline levels and variance. We used a level of significance of $\alpha=0.0038$, in order to adjust for multiple comparisons (Bonferroni correction, 13 biomarkers). We assumed that a 40% difference in mean biomarker level between cases and controls would represent a clinically important difference. By standard calculations for normally distributed data, 90 fatal cases and 270 non-fatal controls will provide 80% power to detect a difference between the two groups. To take into account the possibility of smaller differences, the sample sizes required to detect differences of 40%, 30%, and 20% in biomarker levels are shown

in Table 1. To account for possible dropout/loss to follow-up of 10% of patients, approximately 99 fatal cases and 297 controls are required. In order to find 99 fatal cases, a total of 2475 hospitalized patients would need to be enrolled.

Table 1. Sample size calculation for different effect sizes.

Relative difference	Absolute difference (pg/mL)*	n_1 (non-survivors)**	n_2 (survivors)**
40%*	4,400	90	270
30%	3,300	158	474
20%	2,200	335	1065

*Baseline case: Ang-2 concentrations assumed to be 11,000pg/mL in survivors [44].
**Number required to detect a difference between groups with 80% power at p=0.05/13=0.0038 (Bonferroni correction), two-sided.

Estimates from the Jinja Regional Hospital indicate that approximately 750 children under the age of 5 years are admitted monthly, with 20-30 deaths each month. This implies that accrual of all patients to meet the required sample size will take approximately 5 months.

STATISTICAL CONSIDERATIONS

Study Outcome Measures

Primary outcome

Inpatient mortality will be coded as a binary variable. The level of individual biomarkers at hospital admission in fatal cases versus matched non-fatal controls will be compared using conditional logistic regression models, with adjustment of the statistical threshold for significance for multiple comparisons (Bonferroni correction). Combinatorial approaches using multiple biomarkers to predict mortality will employ multivariable conditional logistic regression models to find the linear combination of quantitative biomarker levels (maximum likelihood estimation) that best discriminate between fatal and non-fatal cases, based on the model *c*-index. In addition, classification and regression trees (CRT) will be constructed to explore variables that best predict survival or death.

Secondary outcomes

The length of hospital stay among survivors, an indicator of disease severity and morbidity, will be correlated with individual biomarkers using non-parametric methods (Spearman’s rho). Linear regression models will be used to explore the predictive value of linear combinations of biomarkers.

The clinical severity score (ordinal variable) will be correlated with individual biomarker levels using non-parametric methods (Spearman's rho). Linear regression models will be used to explore the predictive value of linear combinations of biomarkers.

STUDY PROCEDURES/EVALUATIONS

Study Procedures

For pediatric patients presenting to Jinja Regional Referral Hospital in whom admission to hospital is deemed necessary by an attending physician, the parent or guardian will be approached for consent to participate in the study. If granted, a small volume (1mL) of blood will be withdrawn for processing and storage. A RDT for malaria and whole blood lactate level will be performed at the bedside. Basic demographic and clinical data will be collected from the case admission record, and patients will be followed during their hospital admission. Possible outcomes will include: death, discharge without disability, discharge with disability, abscondment, and loss to follow-up. The length of stay among survivors will be recorded (excluding patients leaving against medical advice). Serum samples will be shipped to the collaborating laboratory in Canada for analysis for biomarkers. ELISA-based commercially-available assays for biomarker levels will be used to quantify biomarker levels.

D. PROTECTION OF STUDY SUBJECTS

RISKS RELATED TO THE STUDY

This is an observational study with no intervention. The main risk associated with the study relates to drawing a blood sample, which will be of minimal volume and will be collected by experienced practitioners. There is no risk of anemia related to removal of such a small volume of blood. The risk of infection will be minimized by careful decontamination of the puncture site using alcohol or chlorhexidine. Pain may be minimized using topical anesthetic, according to the practitioner's judgment.

Study participants will be managed at the Jinja Regional Referral Hospital according to national guidelines and local clinical practices. Outcomes assessed in the study will not pose any risk to the study participants (abstracted from the chart record).

Institutional Review Board

The Makerere University (faculty of Medicine) Research and Ethics Committee (MUREC), together with the Institutional Review Board (IRB) of the University of

Toronto will review this protocol. The Uganda National Council of Science and Technology (UNCST) will also review the study.

No deviation from, or changes to the protocol will be made without prior review and documented approval/favorable opinion from the IRB/independent or institutional ethics committee (IEC) of an amendment, except where necessary to eliminate an immediate hazard(s) to participants, or when the change(s) involve(s) only logistical or administrative aspects of the study (eg, change of monitor(s), change of telephone number[s]).

Subject Confidentiality

Study participant information will be kept strictly confidential. Study data will be accessible only to study personnel. Study data will be stored long-term in files and computer databases in locked offices. Access to databases will be password-restricted, and network security measures will be in place to ensure that information cannot be retrieved by personnel not involved with the study. The study monitor or other authorized representatives may inspect all documents and records required to be maintained by the PI, and the study site will permit access to such records.

Potential benefits of the proposed research to study participants and others:

There is no direct benefit for participation in the study. However, we hope that this study may be an impetus for increased research, which is a potential long-term benefit of the study to the community. If this study documents the clinical utility of peripheral blood biomarkers, this would represent an important finding with the potential to accelerate development of clinical tools to promote child survival through improved diagnosis and prognosis.

Consent

Informed consent will be obtained from the caregivers of all children that will participate in the study. The consent process shall be initiated at the time of enrolment into the study and shall continue throughout the child's participation. The consent will be done in the language that the caregiver best understands. If the caregiver provides consent for study participation, the caregiver will be given the consent forms to sign. A copy of the consent form will be given to the caregiver to keep while a duplicate copy will be kept in the patient's file

For illiterate participants, an independent witness will be present during the informed consent process and will sign the consent form as a witness. The caregivers may withdraw consent at any time throughout the course of the study, and this will be made clear in the informed consent process. All individuals will be informed that there is no requirement to join the study and that standard medical care will remain the same regardless of study enrollment.

If the caregiver chooses not to have their child participate in the study, the case will be turned over to the clinical team on duty for routine care of their condition.

Importance of knowledge to be gained:

New tools to facilitate the clinical care of hospitalized febrile children in Africa could improve outcomes and save numerous lives. If this study documents the utility of host defense biomarkers for diagnosis or prognosis in the major pediatric syndromes that account for the majority of childhood deaths, this would represent an important advance in the global objective of improving child survival. Such findings could stimulate further development of clinical tools to assist in the management of febrile children, which may ultimately reduce mortality among critically ill hospitalized children. Given the ease with which peripheral blood biomarkers can be measured, a biomarker-based diagnostic device could be by minimally trained health care workers, potentially requiring only a fingerprick blood test. Furthermore, ELISA-based assays can be developed into point-of-care tools (e.g., malaria RDTs), allowing them to be incorporated into clinical algorithms in areas with minimal laboratory support. Such characteristics make peripheral blood protein biomarkers an attractive avenue of investigation for use in diagnostic devices that could have broad impact in the most resource-constrained settings, achieving rapid penetration into the most remote or rural settings in sub-Saharan Africa.

CAPACITY BUILDING

This project will build capacity for research and innovation at Jinja Hospital and Makerere University. Four study nurses, additional support nursing staff, and two medical officers will be trained in good clinical practices, research methodology and data management. Training will provide added skills including RDT for malaria and point-of-care lactate measurement. Equipment (Lactate Scout point-of-care instrument for lactate measurement) will remain at Jinja Hospital after the conclusion of the study, providing a valuable tool for improved laboratory diagnostics and patient care. Finally, participation in the study design, implementation, analysis and presentation of data will build capacity among local study team investigators for future research activities.

TIMELINE

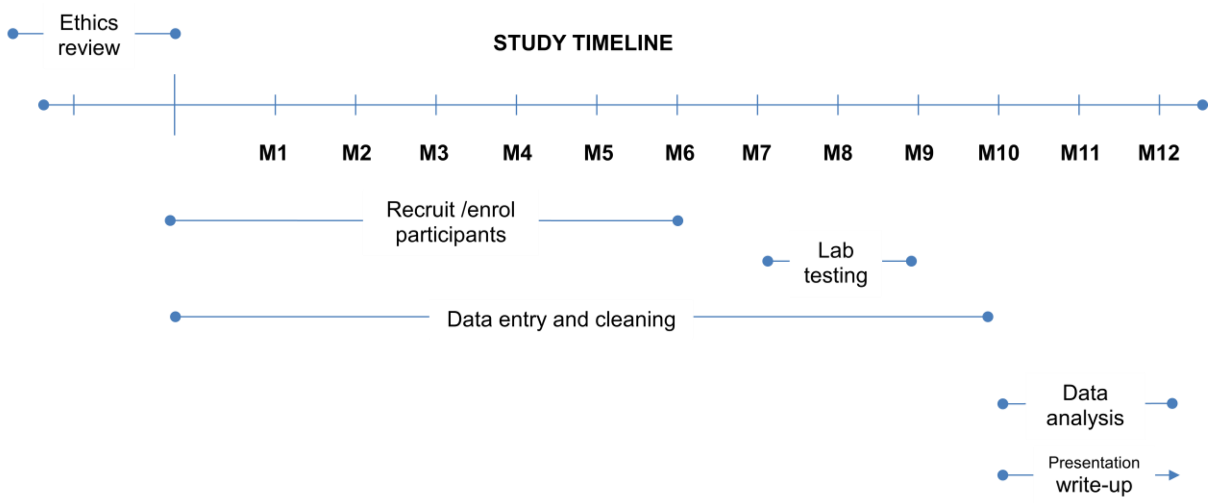
Ethics
review
STUDY TIMELINE
M1
M2
M3
M4
M5
M6
M7
M8
M9
M10
M11
M12
Recruit /enrol
participants
Lab
testing
Data entry and cleaning
Data
analysis
Presentation
write-up

PROPOSED BUDGET

	Item	Description	USD
1.	Personnel	Professional Salaries for study team	14,280
2.	Materials and supplies	RDTs, blood collection supplies, needles, plasticware, standard lab testing and supplies; sample storage and shipping; photocopying and office supplies; communications.	18,000
3.	Equipment	Refrigerator, centrifuge, computer and printer	4,500
4.	Biomarker assays		16,700
5.	Direct Costs	Total direct costs in Uganda	36,780
6.	Indirect Costs		2,940
**Total Costs**			**56,420**

LITERATURE REFERENCES

1.Bawaskar HS. The world's forgotten children. Lancet 2003;361:1224-5.

2.Black RE, Morris SS, Bryce J. Where and why are 10 million children dying every year? Lancet 2003;361:2226-34.

3.Reyburn H, Mbatia R, Drakeley C, et al. Overdiagnosis of malaria in patients with severe febrile illness in Tanzania: a prospective study. BMJ 2004;329:1212.

4.Snow RW, Guerra CA, Noor AM, Myint HY, Hay SI. The global distribution of clinical episodes of Plasmodium falciparum malaria. Nature 2005;434:214-7.

5.Newton CR, Krishna S. Severe falciparum malaria in children: current understanding of pathophysiology and supportive treatment. Pharmacol Ther 1998;79:1-53.

6.Okiro EA, Alegana VA, Noor AM, Mutheu JJ, Juma E, Snow RW. Malaria paediatric hospitalization between 1999 and 2008 across Kenya. BMC Med 2009;7:75.

7.Okiro EA, Alegana VA, Noor AM, Snow RW. Changing malaria intervention coverage, transmission and hospitalization in Kenya. Malar J;9:285.

8.Okiro EA, Hay SI, Gikandi PW, et al. The decline in paediatric malaria admissions on the coast of Kenya. Malar J 2007;6:151.

9.Feng G, Simpson JA, Chaluluka E, Molyneux ME, Rogerson SJ. Decreasing burden of malaria in pregnancy in Malawian women and its relationship to use of intermittent preventive therapy or bed nets. PLoS One;5:e12012.

10.Reyburn H, Mbakilwa H, Mwangi R, et al. Rapid diagnostic tests compared with malaria microscopy for guiding outpatient treatment of febrile illness in Tanzania: randomised trial. BMJ 2007;334:403.

11.Jones G, Steketee RW, Black RE, Bhutta ZA, Morris SS. How many child deaths can we prevent this year? Lancet 2003;362:65-71.

12.Victora CG, Wagstaff A, Schellenberg JA, Gwatkin D, Claeson M, Habicht JP. Applying an equity lens to child health and mortality: more of the same is not enough. Lancet 2003;362:233-41.

13.van der Heyde HC, Nolan J, Combes V, Gramaglia I, Grau GE. A unified hypothesis for the genesis of cerebral malaria: sequestration, inflammation and hemostasis leading to microcirculatory dysfunction. Trends Parasitol 2006;22:503-8.

14.Clark IA, Alleva LM, Mills AC, Cowden WB. Pathogenesis of malaria and clinically similar conditions. Clin Microbiol Rev 2004;17:509-39.

15.Ricciuto DR, dos Santos CC, Hawkes M, et al. Angiopoietin-1 and angiopoietin-2 as clinically informative prognostic biomarkers of morbidity and mortality in severe sepsis. Crit Care Med;39:702-10.

16.Erdman LK, Dhabangi A, Musoke C, et al. Combinations of host biomarkers predict mortality among Ugandan children with severe malaria: a retrospective case-control study. PLoS One;6:e17440.

17.Lyke KE, Burges R, Cissoko Y, et al. Serum levels of the proinflammatory cytokines interleukin-1 beta (IL-1beta), IL-6, IL-8, IL-10, tumor necrosis factor alpha, and IL-12(p70) in Malian children with severe Plasmodium falciparum malaria and matched uncomplicated malaria or healthy controls. Infect Immun 2004;72:5630-7.

18.Chiwakata CB, Manegold C, Bonicke L, Waase I, Julch C, Dietrich M. Procalcitonin as a parameter of disease severity and risk of mortality in patients with Plasmodium falciparum malaria. J Infect Dis 2001;183:1161-4.

19.Armah H, Wilson N, Sarfo B, et al. Cerebrospinal fluid and serum biomarkers of cerebral malaria mortality in Ghanaian children. Malaria Journal 2007;6:147.

20.Bouchon A, Dietrich J, Colonna M. Cutting edge: inflammatory responses can be triggered by TREM-1, a novel receptor expressed on neutrophils and monocytes. J Immunol 2000;164:4991-5.

21.Bouchon A, Facchetti F, Weigand MA, Colonna M. TREM-1 amplifies inflammation and is a crucial mediator of septic shock. Nature 2001;410:1103-7.

22.Chimma P, Roussilhon C, Sratongno P, et al. A distinct peripheral blood monocyte phenotype is associated with parasite inhibitory activity in acute uncomplicated Plasmodium falciparum malaria. PLoS Pathog 2009;5:e1000631.

23.Gomez-Pina V, Soares-Schanoski A, Rodriguez-Rojas A, et al. Metalloproteinases shed TREM-1 ectodomain from lipopolysaccharide-stimulated human monocytes. J Immunol 2007;179:4065-73.

24.Ford JW, McVicar DW. TREM and TREM-like receptors in inflammation and disease. Curr Opin Immunol 2009;21:38-46.

25.Beare NA, Harding SP, Taylor TE, Lewallen S, Molyneux ME. Perfusion abnormalities in children with cerebral malaria and malarial retinopathy. J Infect Dis 2009;199:263-71.

26.Medana IM, Turner GD. Human cerebral malaria and the blood-brain barrier. Int J Parasitol 2006;36:555-68.

27.Dietmann A, Helbok R, Lackner P, et al. Endoglin in African children with Plasmodium falciparum malaria: a novel player in severe malaria pathogenesis? J Infect Dis 2009;200:1842-8.

28.Turner GD, Ly VC, Nguyen TH, et al. Systemic endothelial activation occurs in both mild and severe malaria. Correlating dermal microvascular endothelial cell phenotype and soluble cell adhesion molecules with disease severity. Am J Pathol 1998;152:1477-87.

29.Shapiro NI, Yano K, Okada H, et al. A prospective, observational study of soluble FLT-1 and vascular endothelial growth factor in sepsis. Shock 2008;29:452-7.

30.Yano K, Liaw PC, Mullington JM, et al. Vascular endothelial growth factor is an important determinant of sepsis morbidity and mortality. J Exp Med 2006;203:1447-58.

31.Casals-Pascual C, Idro R, Gicheru N, et al. High levels of erythropoietin are associated with protection against neurological sequelae in African children with cerebral malaria. Proc Natl Acad Sci U S A 2008;105:2634-9.

32.Parikh SM, Mammoto T, Schultz A, et al. Excess circulating angiopoietin-2 may contribute to pulmonary vascular leak in sepsis in humans. PLoS Med 2006;3:e46.

33.Fiedler U, Reiss Y, Scharpfenecker M, et al. Angiopoietin-2 sensitizes endothelial cells to TNF-alpha and has a crucial role in the induction of inflammation. Nat Med 2006;12:235-9.

34.Conroy AL, Lafferty EI, Lovegrove FE, et al. Whole blood angiopoietin-1 and -2 levels discriminate cerebral and severe (non-cerebral) malaria from uncomplicated malaria. Malar J 2009;8:295.

35.Conroy AL, Phiri H, Hawkes M, et al. Endothelium-based biomarkers are associated with cerebral malaria in Malawian children: a retrospective case-control study. PLoS One;5:e15291.

36.Bridges DJ, Bunn J, van Mourik JA, et al. Rapid activation of endothelial cells enables P. falciparum adhesion to platelet decorated von Willebrand factor strings. Blood 2009:blood-2009-07-235150.

37.Boggild AK, Krudsood S, Patel SN, et al. Use of peroxisome proliferator-activated receptor gamma agonists as adjunctive treatment for Plasmodium falciparum malaria: a randomized, double-blind, placebo-controlled trial. Clin Infect Dis 2009;49:841-9.

38.Hawkes M, Opoka RO, Namasopo S, et al. Nitric oxide for the adjunctive treatment of severe malaria: Hypothesis and rationale. Med Hypotheses;77:437-44.

39.Hawkes M, Opoka RO, Namasopo S, et al. Inhaled nitric oxide for the adjunctive therapy of severe malaria: Protocol for a randomized controlled trial. Trials;12:176.

40.Helbok R, Kendjo E, Issifou S, et al. The Lambarene Organ Dysfunction Score (LODS) is a simple clinical predictor of fatal malaria in African children. J Infect Dis 2009;200:1834-41.

41.Taori RN, Lahiri KR, Tullu MS. Performance of PRISM (Pediatric Risk of Mortality) score and PIM (Pediatric Index of Mortality) score in a tertiary care pediatric ICU. Indian J Pediatr;77:267-71.

42.Leteurtre S, Martinot A, Duhamel A, et al. Validation of the paediatric logistic organ dysfunction (PELOD) score: prospective, observational, multicentre study. Lancet 2003;362:192-7.

43.Lacroix J, Cotting J. Severity of illness and organ dysfunction scoring in children. Pediatr Crit Care Med 2005;6:S126-34.

44.Yeo TW, Lampah DA, Gitawati R, et al. Angiopoietin-2 is associated with decreased endothelial nitric oxide and poor clinical outcome in severe falciparum malaria. Proc Natl Acad Sci U S A 2008;105:17097-102.
