# Supplementary material for: Immune and endothelial activation markers and risk stratification of childhood pneumonia in Uganda: A secondary analysis of a prospective cohort study
Source: PLoS Med. 2022 Jul 13;19(7):e1004057. doi: 10.1371/journal.pmed.1004057 (PMC9328519; doi:10.1371/journal.pmed.1004057)
Supplement: S1 Fig — (DOCX) [file pmed.1004057.s003.docx]

| **Supplementary Figure 1.** Flow chart of children included in the analysis by IMCI pneumonia and severe pneumonia. |
| --- |
|  |
| Deaths presented in this figure represent 48 hour in-hospital mortality (primary outcome). There were a total of 63 in-hospital deaths in children with severe pneumonia, 1 in-hospital death in non-severe pneumonia, and a total of 27 in-hospital deaths in children without pneumonia. Abbreviations: IMCI, integrated management of childhood illness. |
