# Supplementary material for: Immune and endothelial activation markers and risk stratification of childhood pneumonia in Uganda: A secondary analysis of a prospective cohort study
Source: PLoS Med. 2022 Jul 13;19(7):e1004057. doi: 10.1371/journal.pmed.1004057 (PMC9328519; doi:10.1371/journal.pmed.1004057)
Supplement: S2 Fig — (DOCX) [file pmed.1004057.s004.docx]

| **Supplementary Figure 2.** Plasma concentrations of immune and endothelial activation markers in children with severe pneumonia who died in hospital compared with those who survived. |
| --- |
| **** |
| Plasma concentration of (a) sTREM-1 (*P* < 0.001), (b) sFlt-1 IL-8 (*P* < 0.001), (c) IL-6 (*P* < 0.001), (d) Angpt-2 (*P* < 0.001), (e) IL-8 (*P* < 0.001), (f) Angpt-1 (*P* < 0.001), (g) sTNFR-1 (*P* < 0.001), (h) sICAM-1 (*P* = 0.024), (i) CHI3L1 (*P* < 0.001), (j) sVCAM-1 (*P* = 0.027), (k) CXCL-10/IP-10 (*P* = 0.910). Abbreviations: Angpt-1, angiopoietin-1; Angpt-2, angiopoietin-2; CHI3L1, chitinase-3-like-1 protein; IL-6, interleukin-6; IL-8, interleukin-8; IP10/CXCL-10, interferon-gamma-inducible protein-10/c motif chemokine 10; sFlt-1, soluble fms-like tyrosine kinase-1; sICAM-1, soluble intracellular adhesions molecule-1; sTNFR-1, soluble tumor necrosis factor receptor-1; sTREM-1, soluble triggering receptor expressed on myeloid cells-1; sVCAM-1, soluble vascular cell adhesion molecule-1. |
