# Supplementary material for: Immune and endothelial activation markers and risk stratification of childhood pneumonia in Uganda: A secondary analysis of a prospective cohort study
Source: PLoS Med. 2022 Jul 13;19(7):e1004057. doi: 10.1371/journal.pmed.1004057 (PMC9328519; doi:10.1371/journal.pmed.1004057)
Supplement: S3 Fig — (DOCX) [file pmed.1004057.s005.docx]

| **Supplementary Figure 3:** ROCs for predicting all in-hospital mortality using sTREM-1 versus common clinical parameters. |
| --- |
|  |
| (a) sTREM-1 (AUROC 0.849, 95% CI 0.799-0.899) compared with lactate (AUROC 0.705, 95% CI 0.664-0.826, *P* < 0.001), respiratory rate (RR) (AUROC 0.622, 95% CI 0.543-0.701, *P* < 0.001), and oxygen saturation by pulse oximetry (SpO_2_) (AUROC 0.658, 95% CI 0.573-0.742, *P* = 0.002) in cases of IMCI pneumonia and (b) sTREM-1 (AUROC 0.843, 95% CI 0.794-0.893) compared with lactate (AUROC 0.682, 95% CI 0.638-0.803, *P* < 0.001), RR (AUROC 0.609, 95% CI 0.58-0.690, *P* < 0.001), and SpO_2_ (AUROC 0.652, 95% CI 0.567-0.737, *P* = 0.002) in cases of severe pneumonia. Abbreviations: ROC, receiver operating characteristics; RR, respiratory rate; SpO_2_, oxygen saturation; sTREM-1, soluble triggering receptor expressed on myeloid cells-1. |
