# Supplementary material for: Immune and endothelial activation markers and risk stratification of childhood pneumonia in Uganda: A secondary analysis of a prospective cohort study
Source: PLoS Med. 2022 Jul 13;19(7):e1004057. doi: 10.1371/journal.pmed.1004057 (PMC9328519; doi:10.1371/journal.pmed.1004057)
Supplement: S4 Fig — (DOCX) [file pmed.1004057.s006.docx]

| **Supplementary Figure 4:** ROCs for predicting all in-hospital mortality using sTREM-1 versus non-specific markers of inflammation |
| --- |
|  |
| a) sTREM-1 (AUROC 0.858, 95% CI 0.808-0.907) compared with PCT (AUROC 0.649, 95% CI 0.569-0.729, *P* < 0.001) and CRP (AUROC 0.564, 95% CI 0.479-0.68, *P* < 0.001) in cases of IMCI pneumonia and (b) sTREM-1 (AUROC 0.852, 95% CI 0.802-0.901) compared with PCT (AUROC 0.641, 95% CI 0.560-0.721, *P* < 0.001) and CRP (AUROC 0.562, 95% CI 0.476-0.647, *P* < 0.001) in cases of severe pneumonia. Abbreviations: CRP, c-reactive protein; PCT, procalcitonin; ROC, receiver operating characteristics; sTREM-1, soluble triggering receptor expressed on myeloid cells-1. |
