# Supplementary material for: Immune and endothelial activation markers and risk stratification of childhood pneumonia in Uganda: A secondary analysis of a prospective cohort study
Source: PLoS Med. 2022 Jul 13;19(7):e1004057. doi: 10.1371/journal.pmed.1004057 (PMC9328519; doi:10.1371/journal.pmed.1004057)
Supplement: S1 Table — (DOCX) [file pmed.1004057.s007.docx]

**Supplementary Table 1:** Plasma severity marker concentrations at hospital presentation

|  | IMCI Pneumonia  (n = 805) | Severe Pneumonia  (n = 616) |
| --- | --- | --- |
| **Inflammatory Proteins** | | |
| sTREM-1 (pg/mL) | 266.9 [174.0, 433.1] | 288.4 [182.6, 483.6] |
| IL-6 (pg/mL) | 50.0 [12.3, 192.0] | 55.6 [13.7, 241.0] |
| IL-8 (pg/mL) | 15.8 [2.5, 55.7] | 18.1 [6.8, 67.7] |
| sTNFR1 (pg/mL) | 3429.2 [2230.1, 6119.9] | 3574.6 [2322.1, 6556.9] |
| CHI3L1 (ng/mL) | 48.0 [21.6, 11.4] | 50.5 [23.2, 130.6] |
| IP-10/CXCL-10 (pg/mL) | 449.5 [178.4, 1296.2] | 441.0 [178.3, 1173.1] |
| **Angiogenic Proteins** | | |
| sFlt-1 (pg/mL) | 453.4 [252.3, 1023.5] | 513.1 [270.1, 1140.9] |
| Angpt-2 (pg/mL) | 9441.8 [5944.7, 15887.7] | 10663.3 [6343.6, 17051.5] |
| Angpt-1 (pg/mL) | 2240.5 [935.2, 5308.0] | 1955.1 [858.1, 5260.7] |
| sICAM-1 (ng/mL) | 748.8 [400.9, 1307.2] | 780.1 [412.5, 1420.1] |
| sVCAM-1 (ng/mL) | 4297.4 [2503.2, 7205.4] | 4449.8 [2546.5, 7344.0] |

^1^Data are presented as median [IQR]. Abbreviations: Angpt-1, angiopoietin-1; Angpt-2, angiopoietin-2; CHI3L1, chitinase-3-like-1 protein; IL-6, interleukin-6; IL-8, interleukin-8; IMCI, integrated management of childhood illness; IP10/CXCL-10, interferon-gamma-inducible protein-10/c motif chemokine 10; sFlt-1, soluble fms-like tyrosine kinase-1; sICAM-1, soluble intracellular adhesions molecule-1; sTNFR-1, soluble tumor necrosis factor receptor-1; sTREM-1, soluble triggering receptor expressed on myeloid cells-1; sVCAM-1, soluble vascular cell adhesion molecule-1.
