# Supplementary material for: Immune and endothelial activation markers and risk stratification of childhood pneumonia in Uganda: A secondary analysis of a prospective cohort study
Source: PLoS Med. 2022 Jul 13;19(7):e1004057. doi: 10.1371/journal.pmed.1004057 (PMC9328519; doi:10.1371/journal.pmed.1004057)
Supplement: S2 Table — (DOCX) [file pmed.1004057.s008.docx]

**Supplementary Table 2:** Area under receiver operating characteristics curve (AUROC) for the outcome of in-hospital mortality for single immune and endothelial activation marker models.

|  | **IMCI Pneumonia (n = 805)** | | **Severe Pneumonia (n = 616)** | |
| --- | --- | --- | --- | --- |
| Biomarker | AUROC  (95% CI)^1^ | P Value^2^ | AUROC  (95% CI)^1^ | P Value^2^ |
| sTREM-1 | 0.885 (0.809, 0.904) | n/a | 0.857 (0.803, 0.898) | n/a |
| IL-8 | 0.791 (0.722, 0.860) | 0.40 | 0.775 (0.704, 0.847) | 0.018 |
| Angpt-2 | 0.784 (0.722, 0.845) | 0.17 | 0.758 (0.693, 0.823) | 0.002 |
| CHI3L1 | 0.754 (0.686, 0.823) | 0.004 | 0.747 (0.677, 0.818) | <0.001 |
| sFlt-1 | 0.750 (0.679, 0.820) | < 0.001 | 0.739 (0.668, 0.810) | <0.001 |
| IL-6 | 0.726 (0.647, 0.804) | 0.005 | 0.722 (0.643, 0.801) | 0.001 |
| sTNFR1 | 0.703 (0.623, 0.784) | <0.001 | 0.699 (0.618, 0.779) | <0.001 |
| VCAM-1 | 0.584 (0.505, 0.664) | <0.001 | 0.592 (0.508, 0.668) | <0.001 |
| ICAM-1 | 0.582 (0.497, 0.666) | <0.001 | 0.577 (0.491, 0.662) | <0.001 |
| IP10/CXCL-10 | 0.467 (0.390, 0.544) | <0.001 | 0.473 (0.394, 0.551) | <0.001 |
| Angpt-1 | 0.345 (0.279, 0.410) | <0.001 | 0.366 (0.298, 0.433) | <0.001 |

^1^Single variable logistic regression performed on log(e)-transformed biomarker values ranked in descending AUROC c-statistic values, ^2^P values represent Bonferroni-corrected AUROC model comparison relative to top biomarker model. Abbreviations: Angpt-1, angiopoietin-1; Angpt-2, angiopoietin-2; AUROC, area under receiver operating characteristic curve; CHI3L1, chitinase-3-like-1 protein; CI, confidence interval; IL-6, interleukin-6; IL-8, interleukin-8; IMCI, integrated management of childhood illness; IP10/CXCL-10, interferon-gamma-inducible protein-10/c motif chemokine 10; sFlt-1, soluble fms-like tyrosine kinase-1; sICAM-1, soluble intracellular adhesions molecule-1; sTNFR-1, soluble tumor necrosis factor receptor-1; sTREM-1, soluble triggering receptor expressed on myeloid cells-1; sVCAM-1, soluble vascular cell adhesion molecule-1.
