# Supplementary material for: Immune and endothelial activation markers and risk stratification of childhood pneumonia in Uganda: A secondary analysis of a prospective cohort study
Source: PLoS Med. 2022 Jul 13;19(7):e1004057. doi: 10.1371/journal.pmed.1004057 (PMC9328519; doi:10.1371/journal.pmed.1004057)
Supplement: S3 Table — (DOCX) [file pmed.1004057.s009.docx]

**Supplementary Table 3:** Area under receiver operating characteristics (AUROC) for the outcome of 48 hour and in-hospital mortality for sTREM-1, respiratory rate, pulse oximetry, lactate, PCT, and CRP.

|  | **IMCI Pneumonia (n = 805)** |  | **Severe Pneumonia (n = 616)** |  |
| --- | --- | --- | --- | --- |
|  | AUROC  (95% CI) | P Value^1^ | AUROC  (95% CI) | P Value^1^ |
| **48-hour mortality** | | | | |
| sTREM-1 | 0.878 (0.832-0.924) | n/a | 0.863 (0.814-0.911) | n/a |
| RR | 0.615 (0.528-0.702) | <0.001 | 0.594 (0.505-0.684) | <0.001 |
| SpO_2_ | 0.685 (0.594-0.776) | 0.002 | 0.673 (0.582-0.764) | 0.002 |
| RR + SpO_2_ | 0.725 (0.647-0.804) | <0.001 | 0.706 (0.626-0.786) | <0.001 |
| Lactate | 0.745 (0.664-0.826) | <0.001 | 0.721 (0.638-0.803) | <0.001 |
| **In-hospital mortality** | | | | |
| sTREM-1 | 0.849 (0.799-0.899) | n/a | 0.843 (0.794-0.893) | n/a |
| RR | 0.622 (0.543-0.701) | <0.001 | 0.609 (0.528-0.690) | <0.001 |
| SpO_2_ | 0.658 (0.573-0.742) | 0.002 | 0.652 (0.567-0.737) | 0.002 |
| RR + SpO_2_ | 0.714 (0.642-0.786) | <0.001 | 0.702 (0.628-0.775) | <0.001 |
| Lactate | 0.705 (0.664-0.826) | <0.001 | 0.682 (0.638-0.803) | <0.001 |
| **48-hour mortality** | | | | |
| sTREM-1 | 0.881 (0.835-0.927) | n/a | 0.856 (0.817-0.914) | n/a |
| PCT | 0.650 (0.566-0.734) | <0.001 | 0.632 (0.547-0.716) | <0.001 |
| CRP | 0.562 (0.472-0.653) | <0.001 | 0.552 (0.461-0.644) | <0.001 |
| **In-hospital mortality** | | | | |
| sTREM-1 | 0.858 (0.808-0.907) | n/a | 0.852 (0.802-0.901) | n/a |
| PCT | 0.649 (0.569-0.729) | <0.001 | 0.641 (0.560 -0.721) | <0.001 |
| CRP | 0.564 (0.479-0.648) | <0.001 | 0.562 (0.476 – 0.647) | <0.001 |

^1^P values represent Bonferroni-corrected AUROC model comparison relative to sTREM-1. Abbreviations: AUROC, area under receiver operating characteristic curve; CRP, c-reactive protein; IMCI, integrated management of childhood illness; PCT, procalcitonin; RR, respiratory rate; SpO_2_, oxygen saturation; sTREM-1, soluble triggering receptor expressed on myeloid cells-1.
